# Lower margins are tied to companies’ climate performance rather than to low-carbon assets

**DOI:** 10.1016/j.crsus.2024.100155

**Published:** 2024-08-23

**Authors:** Marie Fricaudet, Sophia Parker, Nadia Ameli, Tristan Smith

**Affiliations:** 1 UCL Energy Institute, London, UK; 2 UCL Institute for Sustainable Resources, London, UK; 3 London, UK

**Keywords:** climate finance, climate risk, investors’ expectations, climate transitions, shipping

## Abstract

Lenders are likely to face significant financial risks from the shift to a low-carbon economy, but it remains unclear whether such risks are incorporated into their lending practices. The extent of this risk depends on whether banks incorporate such risks into their lending activity and whether financial instruments’ tenors are long enough to cover the period when such risks materialize. Using a case study of shipping loans, we combine quantitative data and semi-structured interviews with key shipping debt providers. Our results show that banks, in particular signatories of the Poseidon Principles, a voluntary disclosure initiative in shipping, have started to price in the climate score of shipowners they lend to after the Paris Agreement but on a corporate rather than an asset basis. However, signatories do not differentiate their margins based on a ship’s carbon intensity, despite a relatively long loan maturity, reinforcing the limitations of disclosure initiatives to influence investment outlays.

## Highlights

• After 2015, banks started rewarding borrowers’ climate performance
• Poseidon Principles signatories now reward borrowers’ climate performance
• Ships’ carbon intensity does not impact their loans’ pricing
• Financial policy and action are needed to align shipping finance with climate goals

## Science for society

This study explores how the climate performance of a borrower and of an asset financed affects the cost of debt by using a case study of shipping loans. The study highlights that although some banks reward companies with high climate scores, they do not distinguish loan terms based on individual ships’ carbon intensity. Furthermore, despite initiatives like the Poseidon Principles, by which lenders disclose the carbon intensity of their shipping portfolio, the expected reduction in financing costs for greener ships has not been observed. The findings suggest that strengthening disclosure initiatives and implementing regulatory measures could better align the financial sector with climate goals. Public financial bodies can also play a crucial role by providing direct support for cleaner assets. By addressing these issues, the study offers pathways to enhance the financial sector’s role in achieving decarbonization, ultimately contributing to global efforts to mitigate climate change.

## Graphical abstract

**Figure:**
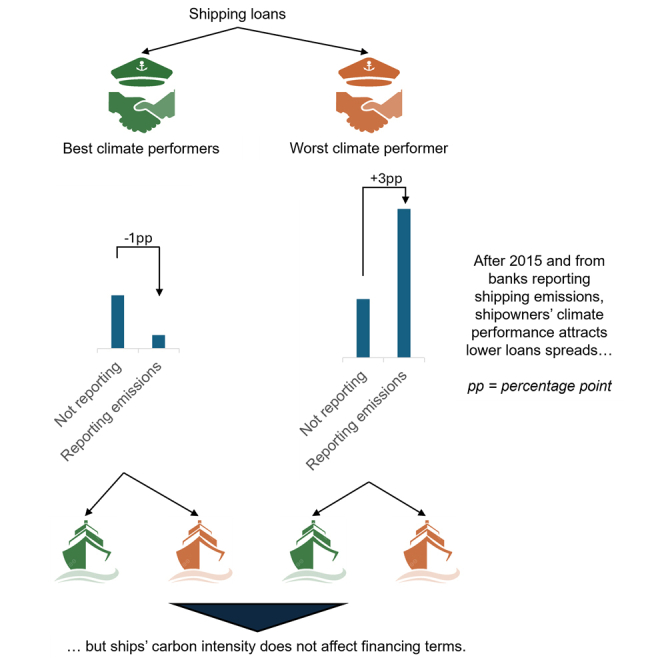

## Teaser

Fricaudet et al. show that banks reward borrowers with high climate scores but do not adjust loans based on assets’ carbon intensity, using shipping as a case study. Even voluntary emission disclosers are not adjusting. This suggests that enhanced disclosure and financial regulation are needed to better align shipping finance with climate goals.

## Introduction

In his speech, the “Tragedy of the horizons,” the Governor of the Bank of England pointed out how climate-related risks might affect a company’s performance.^1^ Climate-related risks might materialize as a consequence of climate-led extreme events (physical risks) and through the stranding of assets, when the introduction of climate policies leads to a sudden and unexpected devaluation of some assets (transition risks).^2^^,^^3^ The latter is particularly relevant for shipping financiers, as ships are long-lived assets (around 20–25 years) largely financed by debt, and a possible asset devaluation could strongly impact the lenders' financing portfolios. Recent evidence shows that a wide range of existing shipping capacity is at risk of being stranded, as the existing fleet is expected to emit 30%–40% more than the allocated shipping carbon budget if they continue operating in current conditions and until the end of their life.^4^^,^^5^

The shipping sector is under growing pressure from governments, climate-conscious investors, and the broader public to decarbonize.^6^ Initial efforts include the International Maritime Organization (IMO) adopting in 2018 an initial strategy to reduce absolute shipping emissions by 50% compared with 2008 levels, by 2050,^7^ a target which member states agreed in 2023 to strengthen to a net-zero greenhouse gas (GHG) emissions target by around 2050.^8^ More recently, the European Union (EU) has included shipping in the EU carbon market.^9^^,^^10^ Shipping customer pressure is also increasing, as demonstrated by initiatives including the Sea Cargo Charter, where signatory charterers commit to report their shipping emissions against a decarbonization trajectory, or where cargo owners of zero-emission vessels, with container customers such as Amazon or IKEA, are committing to using zero-emission shipping from 2025 onward.^11^

In the ship financing community, 34 lenders covering more than 50% of the global ship finance portfolio^12^ have committed to assess and report on the carbon intensity of their shipping portfolio under the Poseidon Principles initiative. Through this framework, the signatories have also claimed to have directly supported the uptake of energy efficiency technologies, for example, by issuing sustainability-linked loans. Such climate initiatives call for greater transparency and climate-related disclosure, the setting of emissions reduction targets, and more climate-aligned strategies.^13^

Despite this increased engagement, it is still an open question whether lenders are accounting for, and internalizing, climate performance (which includes GHG emissions) in their decision process. In this article, we focus on one aspect of environmental performance, namely the action and progress an organization is taking to reduce their GHG emissions (hereto referred to as the “climate performance”). The literature has found contradictory evidence on whether lenders account for a company’s climate performance in the prices of their financial products, particularly the cost of debt. Although some studies find that climate performance has no effect on bond yields,^14^^,^^15^ others suggest that some financial actors have started to incorporate it into bond yields and loan margins, although insufficiently.^16^^,^^17^^,^^18^^,^^19^ In particular, the Paris Agreement could have increased the importance of climate performance as a factor in lenders’ decision making, as debt pricing has begun to reflect borrowers’ climate performance and owned fossil fuel reserves since the agreement.^16^^,^^20^

Traditionally, loan margins are determined by loan characteristics (e.g., collateral, number of lenders, maturity), borrower characteristics (e.g., profitability, leverage, size), and market dynamics.^18^^,^^20^^,^^21^^,^^22^ More recently, there are indications from various sectors that companies’ environmental, social, and governance (ESG) performance, or simply the environmental component, is considered as an additional factor.^16^^,^^21^^,^^23^^,^^24^ The evidence used to make this linkage varies, depending on the borrowers' characteristics, such as the company’s participation in the carbon disclosure project (CDP),^18^^,^^19^ ownership of fossil fuel reserves by borrowers,^20^ reported corporate emissions,^16^ and environmental score.^16^^,^^23^ However, it is not clear from the literature whether lenders also price in the climate performance at the asset level, i.e., the climate performance of the assets they finance.

The shipping lending market offers a unique opportunity to investigate the pricing of climate performance at both the asset and corporate level, as ships are often financed as “secured loans” that use the ship asset as collateral,^25^ but with a recourse on the borrower, i.e., if the loan defaults, shipping banks cannot only liquidate the financed vessels but also claim compensation from the shipowner. In technical terms, “collateral” refers to a lender’s right to possess the asset used as security on a borrower’s potential default or bankruptcy (e.g., the lender reserves the option to liquidate the asset), hence allowing for an exclusive identification between the loan and the underlying asset.

The potential transition risks that ship assets carry may affect ship lenders in two ways. First, like in any other industry, the deterioration of profitability of the companies affected by transition risks can have cascade effects on their lenders by increasing their default rate, which could be amplified by lenders’ interlinkages.^26^^,^^27^^,^^28^ This channel has proven to have a substantial impact on shipping lenders in the past. For example, the oversupply of ship capacity and low shipping earnings following the 2007–2008 financial crisis resulted in a 40% nonperforming loans ratio in the shipping book of German banks, leading to their partial exit from the shipping market and a large impairment in shipping loans.^29^ Second, transition risks could lead to an unexpected devaluation of ship assets due to changes in regulation, technology, or consumer demand. This would impact lenders in the event of a borrower’s bankruptcy because the value of the ship could be the only way to recover the initial amount provided, although ship repossession is, in practice, only used as a last resort.^30^^,^^31^^,^^32^ Ships are still often financed with long-term loans (an average of 7.3 years in our ship finance sample [sample 1], see the experimental procedures section), a period which may potentially expose them to the materialization of transition risks. On the other hand, other types of loans offered to shipowners such as corporate loans are usually shorter (3.4 years in the corporate sample [sample 2], see the experimental procedures section), such that they have less exposure to transition risks.

Here, we investigate first whether the climate performance measured at the asset level and the corporate level are reflected in the lending activity of lenders. We do not investigate the quality of the existing instruments available to financiers to measure climate performance at the corporate level (e.g., the climate score in an ESG metrics) and at the asset level (e.g., carbon intensity), nor their relative quality toward one another; we investigate whether they have an influence on the pricing of shipping loans. The underlying assumption is that if lenders were assessing the climate performance of the assets for the companies they finance as a factor influencing financial resilience, they would incorporate such factors into a higher cost of debt. We test whether this has materialized in the shipping sector as an outcome of the Paris Agreement and sectoral disclosure initiatives, namely the Poseidon Principles. We use an explanatory mixed methods approach^33^ to validate and explore the drivers of the quantitative results with insights from nine semi-structured interviews with major shipping debt providers, together representing 24% of the shipping debt portfolio.

We find that banks have started to price in the climate score of shipowners they lend to since the Paris Agreement but on a corporate rather than an asset basis. In particular, membership to the Poseidon Principles leads lenders to reward companies with the best climate scores with a 4% lower point margin, as opposed to those with the worst scores. However, signatories do not differentiate their margins based on a ship’s carbon intensity.

## Results

### Development of shipowners’ financing costs

The interviews showed that lenders have collected an increasing amount of data on the climate performance (e.g., carbon emissions and air pollution) of their clients and on the assets financed in the last decade, the latter being, however, secondary. However, it is not clear how these data have translated into concrete decisions about loan pricing from the collected qualitative data, as answers from the interviewees were often vague in this respect. According to one interviewee:

The full effect of ESG and climate is not yet included in that model [internal risk rating]. So that is the kind of additional assessment which we do on the outside. So we have [this] in our credit proposal as separate. We have a full ESG scoring, a checklist of more than 70 questions. But we go through all aspects of ESG, including Poseidon scores, including climate targets, including… it is a lot on climate and environment. But in addition to that on shipping, we do a separate analysis in the credit paper on transition risk. Looking at the short-term regulatory risk and how they look to meet CII and EEXI scorings, [and] the Poseidon scores in relation to that, and we also have done separate analysis then on CII. And also then discussing their longer-term transition plan, fleet development plan, etcetera. It is not yet in the quantitative terms included in the risk rating. (Interview 9).

We therefore quantitatively investigate the impact of the climate performance of the borrower and of the asset on the pricing of loans to shipowners.

A large range of tools and metrics are available to capture climate performance at company (e.g., climate score of a borrower)^34^ and asset level (e.g., the carbon intensity of a ship).^35^ The ability of current scores to represent the actual climate performance of a firm is largely debated as they often diverge in the performance they estimate for the same company^34^ and fail to accurately predict future climate performance and emissions,^36^ but given that investors rely on such scores in their operations, the climate score is used as a proxy for the perception of climate performance at the company level.

The CDP offers one of the most comprehensive public databases of climate scores of companies, containing scores for more than 13,000 companies based on their self-reported carbon emissions data and other factors such as governance and engagement. Thus, the CDP’s climate change score (“climate score”) is used as a proxy for the perceived climate performance at the corporate level.

On the asset side, a metric called the energy efficiency design index (EEDI) measures the carbon intensity of the transported work in grams of CO_2_ per ton cargo-nautical miles under as-designed operating conditions (e.g., the ship operated at design speed, in calm water, and fully loaded) for newbuild ships. The metric is used to comply with the IMO's EEDI regulation, which requires ships that attained EEDI to comply with a required EEDI level. The objective of this regulation is to stimulate innovation and technical improvements in design energy efficiency. EEDI data are not made publicly available, and, as such, this study uses the estimated index value (EIV), which serves as a proxy for the EEDI, and, unlike the latter, can be calculated with publicly available data.^37^ This metric is used over the carbon intensity of the ship in real operating conditions because the sample contains a large share of newbuilds and the real operating conditions of a newbuild ship would not be known to lenders. For consistency between newbuild and existing ships, the EIV was used for all vessels. This issue was acknowledged by one interviewee:

New build is really difficult when you do not have [the] Poseidon Principles score or the relevant data for it. (…) Poseidon Principles performance depends a lot on the actual operation. It is not only the design of the vessel, so even if we have second-hand vessels which have operated with the other client, we are aware that even just the ownership change might result in a change of Poseidon Principles score, maybe due to different trading patterns and so on. But we would try to get AER^38^ data, or if it is a newbuild or a second-hand vessel where it is not available, we would try to go via the EEDI^39^. (Interview 13).

To investigate whether climate performance impacts loan margins, we perform an econometric analysis on a new dataset obtained by matching data from syndicated loans from 2010 to 2021 (Dealscan dataset) to related shipowners and ships (Clarksons’ World Fleet Register [WFR]).^40^ The Dealscan database contains financial information on underwritten loans, including loan margins, defined as basis points over the London Interbank Offered Rate (LIBOR) and various loan characteristics. Due to confidentiality issues, the Dealscan dataset does not identify the ship that was financed by the loan as lenders are sometimes unwilling to publicly disclose which ships they have financed and the related financial terms.

The two databases were linked using an algorithm that matches each loan to individual ships based on the qualitative information displayed in the “deal/purpose/tranche remarks” in the Dealscan dataset, which provides useful information on the ships financed, the build date of the ship (from WFR), and the loan issuance date (from Dealscan). In particular, we used the average lag between the date the loan was underwritten, the date when the ship was delivered, and qualitative indications of which ships were financed for each loan in Dealscan to estimate which ships were financed by each loan. This approach allows us to build an original dataset of newbuild ship asset loans.

As a robustness check, a key lender validated the data-matching process on a sample of transactions representing $7.5 billion or 2% of the total underwritten shipping loans (calculated based on the portfolio of the top 62 shipping banks in 2021^41^). This lender confirmed that the algorithm uniquely matched almost all transactions with the respective ships (90%), showing the validity of the approach. The details of the loans-ships matching algorithm are presented in the experimental procedures section.

The loan margin mainly depends on loan-, lender-, borrower-, time-, and country-specific variables. The conditions of the loan and the financial characteristics of the borrower impact the price of the loan, as they are generally considered proxies for the potential risk that the borrower defaults.^20^^,^^21^^,^^22^^,^^42^ Most shipping loans in our dataset are recourse loans (see details in the experimental procedures section), that is, if the loan defaults, shipping banks have the option to not only liquidate the financed vessels but also to use the borrower’s other assets or income to recover the remaining amount. As a consequence, lenders place great importance on the financial strength of not only the collateral but also the borrower.^43^ We therefore included financial information on the borrower from Refinitiv-Eikon (profitability, size, and leverage). Time and country dummy variables further control for unobserved variables, e.g., the health of the market, which might also affect the riskiness of the loan. Because it is not clear from the literature the factors driving loans dedicated to financing ship assets, the weighted average least-squares estimator (WALS) method is used to determine the best model specification in the absence of a theoretical model.^44^ The initial list of variables was compiled by including traditional drivers of margin identified in the literature and additional variables that were suggested by interviewed lenders (e.g., the second-hand market price index of ships). Finally, as newbuilds exhibit a distinct risk profile compared with second-hand assets and also receive a higher priority ranking in the credit system, we include a control for the financed ships’ age.

### Pricing of the climate performance of the companies’ and ships’ assets

**Figure 1:**
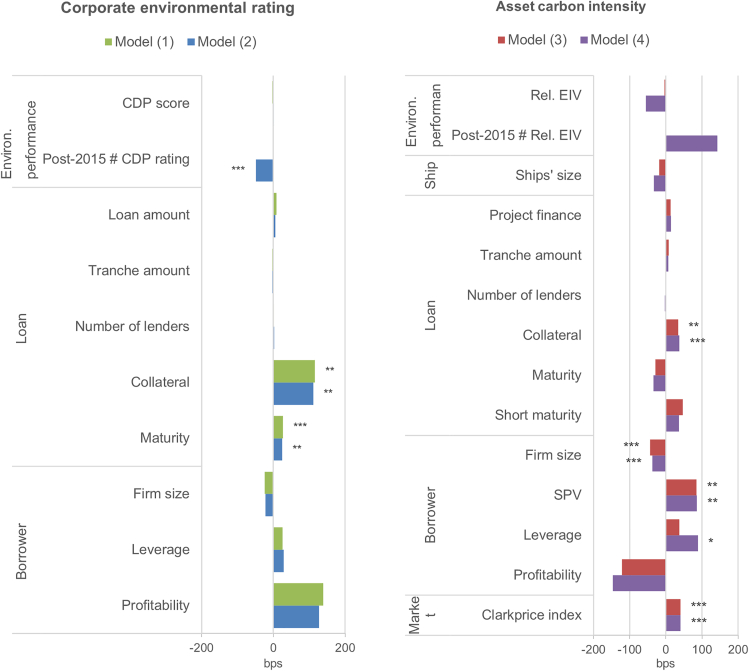
Corporate and asset climate performance and cost of debt The dependent variable is the loan margin. The regression coefficients plotted are estimated using the ordinary least squares (OLS). The carbon disclosure project climate change score is expressed as scores ranging from (highest) to (lowest) and was coded from 0 (lowest) to 8 (highest). The estimated index value (EIV) is normalized by the average EIV of the cohort. Further controls of shipping segment (only in models 3 and 4), loan purpose, repayment type, borrower country, and year fixed effects are included in the models (see details results in the experimental procedures section). Estimates with robust standard errors clustered at the borrower company level. ∗*p* < 0.1, ∗∗*p* < 0.05, ∗∗∗*p* < 0.01*.*

The Paris Agreement was a catalyst for increased ambition from the international finance community to align financial flows with climate priorities. This has led to increased pricing of climate performance on the cost of debt by lenders at the corporate level (Figure 1).

**Figure 2:**
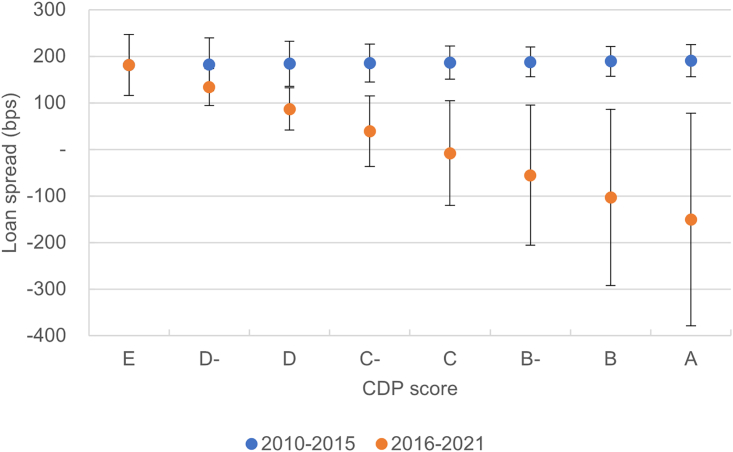
Company’s climate score, Paris Agreement, and the cost of debt Effect of the dependent variable CDP score on the cost of debt before and after 2015, estimated using model (2 in Figure 1 with 95% confidence intervals. The carbon disclosure project (CDP) climate change scores were coded from 0 to 8 with 0 being the lowest possible climate score and 8 the highest.

Companies with a high climate score attracted similar margins as companies with a low climate score over the whole time period of the sample (Figure 1, model 1). However, there is a clear increase in pricing after the Paris Agreement, which indicates that lenders have begun to price the climate score of companies into the cost of debt (Figure 1, model 2). We observe this shift by including in model 2 an interaction term between the companies’ climate score and a post-2015 dummy to capture the shift in pricing of corporate climate performance after the Paris Agreement—and by breaking the periods into pre- and post-Paris Agreement (see the experimental procedures section, models 7 and 8). As a consequence, borrowers with higher climate performance started to attract lower margins only after the Paris Agreement (Figure 2).

**Figure 3:**
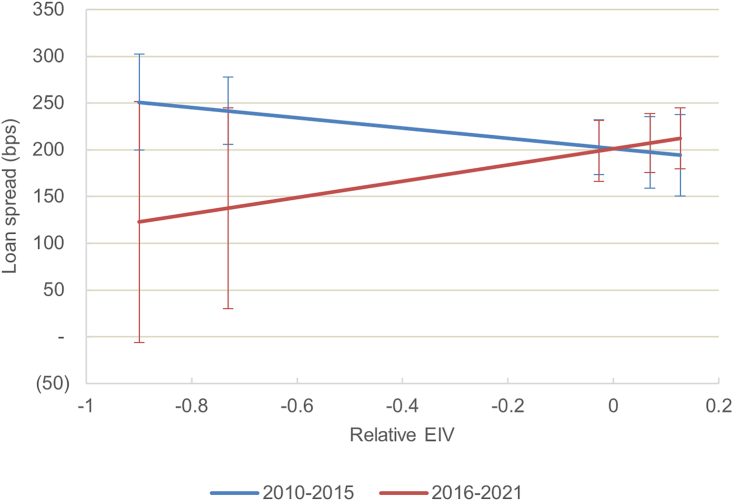
Carbon intensity of the financed ship assets, Paris Agreement, and the cost of debt Effect of the dependent variable of the intensity of the carbon intensity of the ship on the cost of debt before and after 2015, estimated using model 4 with 95% confidence intervals. The relative carbon intensity is the estimated index value (EIV) of financed ships compared with the average EIV of their year cohort. The predictions of the costs of debt were estimated for the 10th, 25th, 50th, 75th, and 90th percentiles of the relative EIV.

However, carbon-intensive ships have attracted a similar cost of debt compared with their counterparts over the entire period (Figure 1, model 3). Interestingly, before the Paris Agreement, carbon-intensive ships were more generously priced (Figure 3), although the result is not significant, and robust across various specifications (see the sensitivity analysis in the supplemental information). A negative carbon intensity is counter-intuitive and would suggest that not only were lenders not favoring climate performance—at least before the Paris Agreement—but that they would also see carbon efficiency as an unnecessary cost and source of risk. From this, it appears that the carbon intensity of ships is at best ignored by lenders and at worst preferred prior to 2015. There is no strong evidence of an evolution in pricing after the Paris Agreement, as the insignificant coefficient of the interaction term between the EIV and a post-2015 dummy in Figure 1, model 4, suggests.

In addition, smaller borrower size is associated with higher margins, indicating increased risk on the loan. Furthermore, high-risk transactions that need to be secured with collateral have, on average, a cost of debt 0.3%–1.1% higher (Figure 1, models 1–4). For corporate financing, an increased maturity attracts higher margins (Figure 1, models 1 and 2), but these do not appear to have a large impact on the cost of debt for ship finance only (Figure 1, models 3 and 4). Surprisingly, a bullish second-hand ship market and higher profitability increase the loan margins (Figure 1, models 3 and 4). This might be because, given the cyclical nature of the shipping industry, lenders expect grim future economic conditions when the market is high, and inversely. Another explanation would be that, during periods of high demand in the shipping market, shipowners place orders for new ships, which increases the demand for loans. Consequently, as demand rises (with the supply remaining relatively constant in the short term), banks are able to charge higher margins.

The shipowners interviewed highlighted that the margins are not driven by the carbon intensity of the ships they financed, nor by climate-related credit risk analysis, but mainly by competition between lenders for a few top-tier shipowners. In fact, the LIBOR margin is set at a minimum above the lender’s capital cost and the loan credit risk, whose calculation excludes any asset-related climate performance. This credit risk, which has not evolved significantly in the last decade, mostly uses backward-looking variables, such as the company’s leverage and profitability, and expected earnings (which do not include carbon costs) based on the historical performance of the asset’s shipping segment. Even when commercial banks mentioned using forward-looking scenarios, it was not specific to a shipping decarbonization scenario.

The lenders interviewed confirmed that the use of this credit risk methodology is a barrier to pricing climate performance, reinforcing an inertia to change it: “The capital requirements for our banks are based on our internal risk rating model. We are a so-called IRB Bank [using an] internal rating-based model approved by the financial regulator (…). We cannot just change that model all the time. (…) But the full effect of ESG and climate is not yet included in that model.” (Interview 9).

However, it appears that some financiers are adapting their heuristics, evidenced by the fact that most of the lenders interviewed have developed tools to measure companies’ climate performance and environmental strategy over the past decade. Some shipping lenders include such company scores in the credit risk analysis, which might explain the positive pricing of the company’s climate score after 2015:

The pricing is still completely risk return driven. (…) What you see now, if you have ESG, [is that] there are certain corporate facilities, but we see it more on the corporate facility basis. If you are, as a company, much more CO_2_-efficient, then you can get slightly lower pricing. Or the other way around, you will be priced higher. (…) On the individual basis with ship finance in bilateral financings, which we do, there is no pricing differentiation yet. So it’s more a selection, a method, you just don’t do this asset anymore. (Interview 5).

This indicates that the climate performance of a company influences pricing at the corporate level but not the asset level. At the asset level, banks are using climate performance of assets as a financing criterion.

### Effect of lenders’ reporting commitments on margins

Whether lenders price the corporate and/or asset climate performance in loan margins might reflect emissions disclosure efforts. The Poseidon Principles allow us to investigate the impact of voluntary disclosure initiatives of lenders on the pricing of climate performance because it is the first sector-wide alignment disclosure agreement with global coverage. We do so by including a dummy variable “Poseidon Principles” in the model, which takes the value 1 if the lender had already signed up to the Poseidon Principles when the loan was issued.

**Figure 4:**
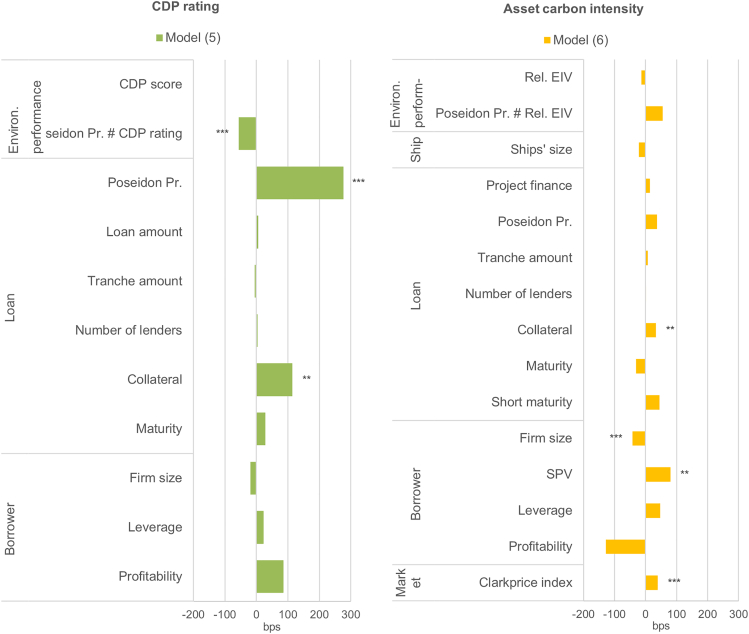
The role of lenders’ commitments on the pricing of climate performance The dependent variable is the loan margin. The regression coefficients plotted are estimated using the ordinary least squares (OLS). Further controls of loan purpose, repayment type, shipping segments (only in model 6), borrower country, and year fixed effects are included in the models (see details results in the experimental procedures section). Estimates with robust standard errors clustered at the borrower company level. ∗*p* < 0.1, ∗∗*p* < 0.05, ∗∗∗*p* < 0.01. The Poseidon Principles is a dummy variable that takes the value 1 when the lender has signed the Poseidon Principles, 0 otherwise. The relative EIV is the annual efficiency ratio compared with the years’ cohort; the CDP is the carbon disclosure project climate change score.

**Figure 5:**
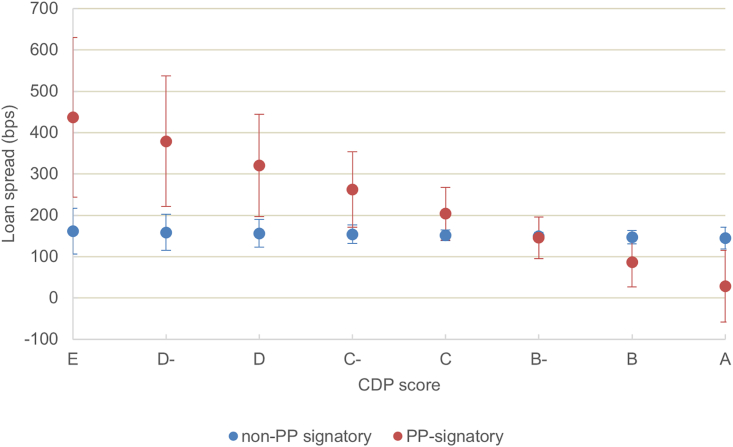
Lenders’ carbon reporting commitment, company’s climate score, and cost of debt Effect of the dependent variable CDP score on the cost of debt estimated when lenders are Poseidon Principles (PPs) signatories (red) and non-signatories (blue). The margins were estimated using model 5 with 95% confidence intervals*.*

**Figure 6:**
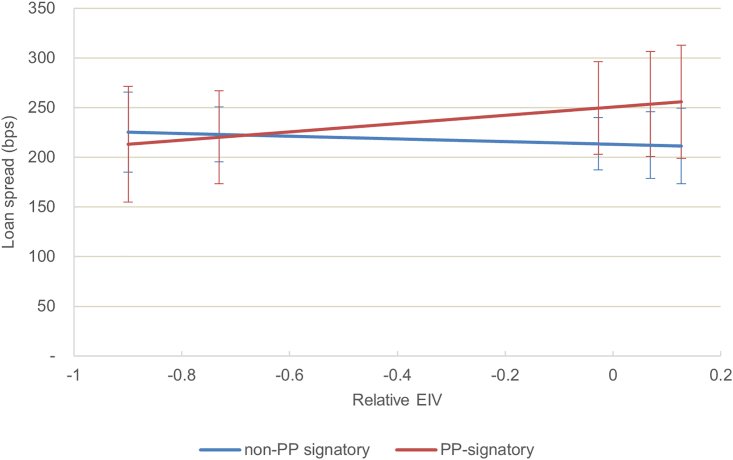
Lenders’ carbon reporting commitment, financed ship assets’ carbon intensity, and cost of debt Effect of the estimated index value (EIV) on the cost of debt estimated when lenders are Poseidon Principles (PPs) signatories (red) and non-signatories (blue). The margins were estimated using the model 6 with 95% confidence intervals. The predictions of the costs of debt were estimated for the 10th, 25th, 50th, 75th, and 90th percentiles of the relative EIV.

The Poseidon Principles have a positive effect on the pricing of the company’s climate score (Figure 4). The scale of this effect is economically significant: the lowest-performing companies face a cost of debt 4% points higher than the highest-performing companies (Figure 5). However, the Poseidon Principles have a negligible effect on the pricing of the ship asset carbon intensity (Figure 4, model 5; Figure 6). This suggests that the voluntary commitment to disclose its financed carbon emissions can have a concrete impact on investment decisions but is not ultimately reflected in the assets financed.

These results suggest that the climate commitments of lenders have translated into an increase in the price of the company’s climate score, but not of the climate performance of the asset. Awareness of the necessity of the transition to low-carbon shipping had a concrete impact on lenders’ behavior, as they provided preferable conditions to shipowners with a higher climate score. This is a clear incentive for borrowers to improve their climate scores and to be perceived as a more sustainable company. However, the results also suggest that even climate-proactive lenders are not aware of the cascade effects of transition risks from the assets to their profitability, as they do not factor the transition risks of their assets into the pricing of the loans they provide.

All interviewed signatories highlighted that the Poseidon Principles had induced a large change in the lenders’ activities, so data related to the carbon intensity of ships were collected and scrutinized systematically in the investment decision process. Some emphasized that they would take into account the climate performance of ships to engage with the client, but the impact on actual decisions and even more on pricing is so far limited: “We use the Poseidon Principles to have a dialogue with their clients. [In respect of] CO_2_, it’s not that we won't finance a ship which is above the pathway, but we want to (…) understand from the owner what they [want and] what their decarbonization strategy is.” (Interview 4). How these metrics impact the decision was not clear and appeared to be an addition to the loan assessment process, while having no effect on the calculated credit risk and therefore the pricing: “I wouldn’t say that banks are pricing ships lower if they’ve got a good AER [annual efficiency ratio, a measure of the ship’s operational carbon intensity] and worse [if] it's got a bad AER. I don't think we've reached that basic situation yet.” (Interview 2).

## Discussion

Shipping lenders were notably misaligned with the efforts of the shipping industry to decarbonize prior to the Paris Agreement when extending loans to shipowners. This study shows that they were either not aware of—or possibly just not prioritizing—transition risk, as they have provided preferable margins to carbon-intensive ships while penalizing those with more efficient designs. These findings are aligned with those of Bell et al.,^45^ who find no evidence that lenders priced the energy intensity of UK homes into the cost of debt before 2018 and instead showed a small premium for energy efficient assets.

However, the lenders’ appetite for climate performance has increased after the Paris Agreement and there are signs that this positively impacted the pricing of the shipowners' climate performance. These results are consistent with those of Seltzer et al.^16^ and Chava,^21^ who show that firms with a higher corporate climate performance pay a lower cost of debt, especially after the Paris Agreement, and those of El Ghoul et al.^23^ and Rojo-Suárez and Alonso-Conde,^24^ who have found similar results for equities, namely on the estimated cost of equity capital and on the stocks betas, respectively. This increased appetite does not mean that lenders are now aware of the transition risks, as those are unlikely to be relevant to the short-term loans that they on average provide to borrowers. Furthermore, this increased appetite is not sufficient as it does not lead to a differentiated margin based on the carbon intensity of the ship. Ships, on the other hand, are often financed by longer-term loans (more than 7 years on average) and would be concerned by transition risks. So, although lenders pay attention to climate scores at a corporate level, they are not yet directly supporting lower-emission ships through a pricing mechanism. This is explained by the lack of formally including asset-level climate performance in the credit risk assessments conducted by major shipping lenders, which are, instead, based on backward-looking metrics of shipowners and the past performance of a shipping segment. This is also linked to the fact that most of shipping debt, at least in our sample, uses the borrower as recourse, so that the importance of the borrower in the risk analysis prevails over the importance of the asset. Furthermore, pricing is explained by competition between banks for clients.

To price in climate-related risks, lenders might want to reward investment in low-carbon assets, and vice versa, not only investments to companies with a strong climate performance. Indeed, companies with a high climate score might not necessarily invest in low-carbon assets. Anecdotal evidence suggests that companies with high climate scores are not more likely to issue green bonds than less environmentally friendly companies,^46^ and companies that have a higher environmental score pollute as much as competitors with lower scores.^47^ Furthermore, when there is collateral, the climate-related risk at the asset level matters, as the collateral is the first way for the bank to recover their money in case of default.^32^^,^^48^ In case of recourse loans, which make up most of our sample, if the collateral is at risk but the rest of the borrower’s fleet is not, this might be less of an issue as the lender can claim the losses against the borrower, hence the need to assess climate-related risk at the corporate level. However, a borrower—even one with a good climate performance—who invest in assets at risk of being stranded going forward would put itself at risk and, consequently, the loans on recourse. Therefore, the lender might still want to incentivize investments in less climate-risky assets.

The ability of lenders to use adequate metrics to measure climate performance and climate-related risks is a critical issue for two main reasons. First, given the lifespan of a ship (20–25 years) and the average tenor of the observed loans (7 years on average for ship finance), the viability of the shipping loans in the lenders' portfolio could likely be at risk in the coming years if the transition risks materialize. Second, in practice, lenders are not yet incentivizing the uptake of carbon-emission ships by lowering the cost of debt; in fact, before 2015, they might have been disincentivizing it.

These results imply that stronger regulation and enforcement action is needed to change investment decisions. Not only is the negative externality of shipping emissions not internalized but also market forces that regulators could have assumed were driving efficiency improvement over time (e.g., lower margins for low-carbon ships as a means to reduce operating costs) are not evidenced in practice for financiers. Using effective policy measures that are clearly aligned to the goals of the Paris Agreement is needed. Examples include a carbon price, stricter performance standards for newbuilds and/or second-hand ships, and subsidies to alternative fuels production, bunkering, and ships.

Our results therefore further contribute to the existing literature that questions the relevance of the current metrics used by financiers to measure climate-related risks. Riedl^49^ and Thomä and Chenet^50^ argue that because they rely on backward-looking metrics, these measures are ill-suited to capture transition risks that have not materialized in the past, which, instead, would require forward-looking risk assessments. Our analysis itself is limited in this regard, as both the CDP and the carbon intensity of the ship are backward-looking. For example, the carbon intensity of the ship does not include the possibility and expected cost of adopting cleaner technologies in the future through retrofitting the ship for alternative fuels and energy efficiency measures or the use of drop-in biofuel.

Our analysis further contributes to the nascent evidence on the limited effectiveness of voluntary disclosure initiatives in changing investment outlays.^51^ The Poseidon Principles have not induced a reduced cost of debt from investing in low-carbon assets thus far. This could be related to its recent implementation and the fact that shipping markets have been affected by the consequences of the COVID-19 pandemic, which led to a preference for companies with a higher climate score. The fact that lenders price in the climate score of the borrower means that they might be indirectly promoting low-carbon ships if shipowners with a higher climate score were financing more carbon-efficient ships. However, there is no guarantee of this outcome.

Our analysis further reinforces the argument for strengthening disclosure initiatives and intensifying monitoring efforts, or implementing more interventionist policies to regulate the financial sector that are not solely focused on the emitters’ (here shipowners’) side. Several policy options are available to regulators, ranging from mandating lenders to assess and disclose emissions financed according to industry standards and imposing taxes on financial actors based on the emission intensity of their portfolios^52^ to adjusting capital adequacy requirements for carbon-efficient (intensive) portfolios via a green supporting factor or implementing green monetary easing policies.^53^^,^^54^^,^^55^ When possible, for example, in project finance or with the use of collateral, such regulations should not only cover the emissions of companies but also the asset financed. Furthermore, direct support from a public financial body can both compensate for the lack of support from existing lenders to finance cleaner assets, for example, through the provision of guarantees.^56^ Export credit facilities are already commonly combined with senior-secured loans in the shipping industry to reduce the cost of capital, and these could be used specifically to facilitate the uptake of cleaner technologies.^56^ Direct support for public financial institutions not only supports low-carbon technologies but also creates a signaling effect of trust that encourages existing lenders to support new technologies.^57^^,^^58^ Those financial interventions and policies would allow the cost of debt to be linked to the climate performance of the borrower and of the asset directly and hence drive the financial system to contribute to the transition to a low-carbon economy.

Our study has several limitations. First, there are various metrics to measure the climate performance of companies and ship assets, which are often only lightly correlated: the environmental scores between rating agencies have an average correlation of 53% in Berg et al.’s sample of metrics,^34^ while credit ratings are correlated at 99%. To address this limitation, we have conducted a sensitivity analysis of the results with alternative measures of climate performance (see the supplemental information). However, the multiplicity of instruments and the lack of coherence between them, especially on the corporate side, highlights the lack of adequate tools to measure the climate performance of borrowers and assets, which is likely an obstacle for pricing climate performance.^34^^,^^59^

Second, the sample used to conduct the asset-level analysis focuses on senior-secured loans per asset. Although this is a common business case, it might not be representative of the whole industry, as the borrowers are generally top-tier clients who own a larger fleet than the industry average (see the analysis on sample bias in the supplemental information). We have, however, controlled for the size of the borrower in the model to remove the bias on the coefficient linked to climate performance, but we have not been able to test whether lenders price in the climate performance in other types of transactions. However, there is not a strong rationale for a difference in the pricing of secured loans made to top-tier borrowers and other secured loans for non-top-tier borrowers. The size of our sample is limited to the available data and is small—especially the ship finance sample (sample 1). This analysis might, therefore, benefit from renewed analysis once further data become available, especially those related to the analysis of the impact of the Poseidon Principles. As those are recent, they cover fewer observations in the samples, and it might take some time for them to have an impact on the behavior of the banks.

## Experimental procedures

### Resource availability

#### Lead contact

Further information and requests for resources should be directed to Marie Fricaudet (m.fricaud@ucl.ac.uk).

#### Material availability

This study did not generate new unique reagents.

#### Data and code availability

Restrictions to the availability of the data used in the econometric analysis (Fuel Use and Emissions [FUSE], Refinitiv-Eikon, Dealscan, Clarksons’ WFR, and Clarksons’ SIN) apply as they are under license for the current study and so are not publicly available. The CDP scores are publicly available and can be found at: https://www.cdp.net/en/companies/companies-scores.

### Quantitative data collection

Borrowers in Dealscan were matched to shipowners in Clarksons’ WFR by the website provided (when available), the stock exchange name (when listed and provided), or their name. Capital-intensive assets like ships can be financed through project finance, i.e., Most likely, in corporate finance, the margin is determined based on the risk assessment of the company, not solely on the project (asset, ship, the collateral).

### Choice of variables to represent climate performance

Let us first look more closely at the variables used to proxy the climate performance. The perceived climate performance of the company is proxied by the CDP climate score. The authors do not intend to suggest that the CDP score, nor any environmental score metrics, is a good representation of the climate performance associated with a company. There is mixed evidence in the literature on the ability of such instruments to predict future company’s climate performance. Although the Kinder, Lydenberg, Domini (KLD), now Morgan Stanley Capital International (MSCI), net environmental score does predict future pollution levels and regulatory penalties, its explanatory power is lower than those of lagged emissions, which suggests that it is not optimally aggregating historical data. ^36^ Furthermore, the environmental scores diverge between the rating agencies, mainly due to the divergence in measurement, which casts doubt on the reliability of the results.^34^ However, the objective of this paper is to study the impact of the perception of climate performance on the pricing of loans rather than the actual performance, so the choice of proxy for the perceived climate performance at the company level was driven by its use and perceived quality by financiers rather than by its actual precision. The latter driver is ignored in the analysis. Given these considerations, the CDP is chosen as a proxy for perceived climate performance at the company level for several reasons. First, the CDP offers one of the most comprehensive public databases of companies' climate scores, containing scores for more than 13,000 companies based on their self-reported carbon emissions data and other factors, such as governance and participation. Second, it is widely and freely available to lenders and is one of the oldest to be published, while other metrics are costly. Third, in 2022, surveyed investors ranked the CDP as the most useful (second most useful in 2018) and second most reliable rating (same as in 2018) in a sample of 13 leading ratings, including Sustainalytics, S&P, and Bloomberg ratings, for example, sustainability.^60^ CDP scores are initially expressed from A (highest score), A, B, B− to E (lowest); they were coded from 0 (lowest) to 8 (highest) for the purpose of the regression. We test the robustness of our approach by using an alternative proxy for perceived climate performance, i.e., a combined indicator equal to the Refinitiv environmental score—Refinitiv environmental controversies score. It is worth noting that this rating was ranked much lower by investors in terms of both usefulness and quality, so results should be taken with caution.^60^

The climate performance of each ship is proxied by its carbon intensity. Specifically, the EIV is used as the standard measure of the ship’s carbon intensity. The EIV is an approximation of the EEDI and measures ships' design carbon intensity while ignoring the operation of the ship. Fleet efficiency has increased over time as a result of high fuel prices rather than regulation, ^61^ so that younger ships are on average more energy efficient than older ones.^62^ In addition, larger ships have on average a lower carbon intensity than smaller ships; the ship type has a large impact on the carbon intensity.^62^ As a result, the carbon emissions of the ship per deadweight are highly dependent on the type of the ship, i.e., whether it transports passengers or commodities and, in the latter case, which cargo is transported, as well as on the size of the ship, so using directly might bias the results. To control for these variations, the difference in the carbon intensity for each ship relative to its cohort was used as a proxy of the ship’s climate performance rather than its absolute carbon intensity, as follows:

(Equation 1) $$CI_{i}=\left({EIV}_{i}-{EIV}_{cst}\right)/{EIV}_{cst}$$

with ${EIV}_{i}$ the carbon intensity of the ship that is part of the peer group defined by ship type c, size bin s, and built in year t. Finally, because more than one ship could be associated with a loan, the climate performance of a loan is computed as the average carbon intensity of its associated ships.

We control the robustness of the results to the choice of metrics by running the model using two alternative metrics. First, shipowners have to measure the annual efficiency ratio (AER) of the ship above 5,000 gross tonnage to comply with the Data Collection System (DCS) introduced by the IMO. This indicator measures the CO_2_ emissions of a ship, divided by the product of its capacity and the distance sailed per year, thus capturing climate performance at asset (ship) level. Since the introduction of the Poseidon Principles, the AERs of the ships are widely collected and scrutinized by shipping lenders, as acknowledged by the interviewees. As DCS data are confidential to the shipowner and requires the consent of the shipowner to be shared, estimated data for EIV/AER were taken from the UMAS FUSE model, which uses satellite and terrestrial AIS data to calculate speed, fuel consumption, and CO_2_ emissions.^63^ A summary of the different metrics used to measure the ships’ carbon intensity can be found in Table 1. Second, we look at the effect of having energy-saving technologies installed onboard ships on the margins. We include a variable that corresponds to the share of ships financed by the loan that are equipped with one energy-saving technology on the ship, as indicated in Clarksons’ WFR. In our sample, those include propeller ducts, rudder bulbs, propeller boss cap fins, and wake equalizing duct. The results of this sensitivity analysis are presented in the supplemental information.

**Table 1:** Description of the metrics to measures ships’ carbon intensity

Metric	Unit	Description	Reference for detailed calculation
EEDI	gCO_2_/ton-nm	design technical carbon intensity at the start of a ship’s life under specific EEDI trial assessment conditions	MEPC.245(66)^64^
EIV	gCO_2_/ton-nm	design technical carbon intensity. It is a simplified form of the EEDI that can be calculated on the basis of publicly available data.	MEPC.215(63)^65^
AER	gCO_2_/dwt-nm	operational carbon intensity, ignoring the utilization of the ship. It is equal to the ratio of carbon emissions over a year divided by the distance traveled over that year and the deadweight.	Poseidon Principles, 2023^66^

Our empirical analysis focuses on syndicated loans that have been provided between 2010 and 2021 to companies that own at least one ship. Our transaction data for the loans are sourced from Dealscan, which collects information on underwritten loans. This database provides various information on the loans, including all-in-spread-drawn (AISD), the lenders, tranche amount, loan conditions (repayment type, tenor, etc.), and the borrower. We select the subset of this database where the borrower owns at least one ship by matching the borrowers to the shipowners on Clarksons’ WFR database, which provides information on the ships owned and the shipowners. This overall dataset of loans awarded to shipowners over the period includes 18,747 observations corresponding to 808 combinations (unique borrower, deal amount, date of deal), called “deals” in the rest of the article. There are more observations than deals in the dataset because one deal is often divided into tranches with differing loan conditions and each tranche is financed by several lenders. One lender × tranche combination constitutes one observation.

Shipowners can borrow money through corporate finance to finance various purposes (e.g., general purpose, takeover, restructuring), not only to finance ships. The climate performance of the borrower might have an influence on the pricing of any of those types of loans; however, we can only measure the impact of the climate performance of the ship when the link between the loan and the asset is clear, i.e., in the case of ship finance. We therefore distinguish between two overlapping samples:

• A ship finance sample (sample 1) that only includes loans whose purpose is specified as “ship finance,” and where the ship(s) financed could be identified (see next section for more information), using a non-recourse structure, typically a special-purpose vehicle (SPV), or via traditional recourse loans. SPVs are included in the sample and identified as they were marked as “special-purpose co,” “project, special-purpose co,” or “infrastructure SPV” in the Dealscan borrower type.
• A corporate finance sample (sample 2) that includes any loans given to shipowners, no matter their specified purpose, and where the borrower had a CDP score. However, project finance loans and loans raised through an SPV are excluded from this sample.

**Figure 7:**
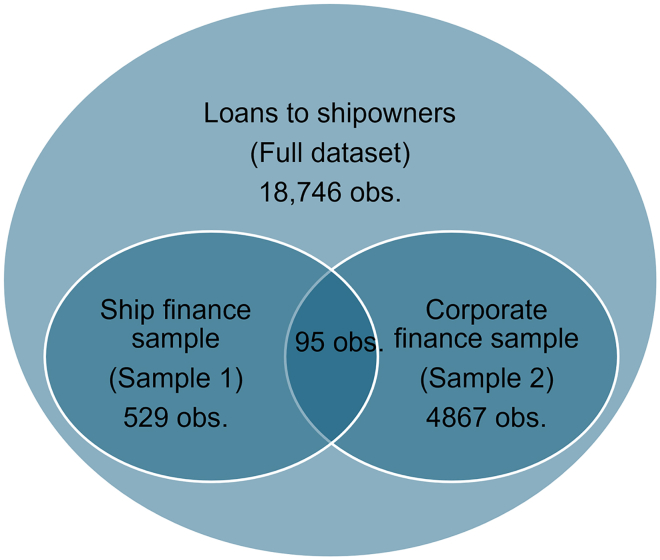
Observations per sample Counts are shown after removing the observations where borrower-related financial information (e.g., leverage, profitability) was missing, as they would be excluded from the regression in Stata.

We use those samples to test the pricing by lenders of the perceived climate performance of the company (sample 2) and of the perceived climate performance of the asset, i.e., the ship (sample 1). The sizes and the overlap of the samples are shown on Figure 7.

**Figure 8:**
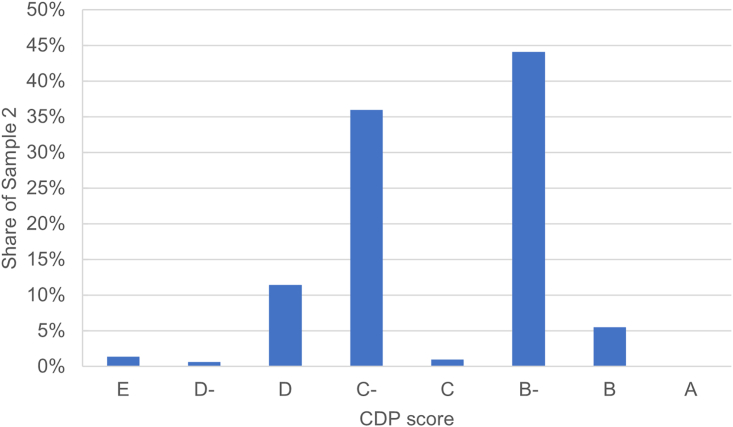
Distribution of loans by CDP score in sample 2 Counts are shown after removing the observations where borrower-related financial information (e.g., leverage, profitability) was missing, as they would be excluded from the regression in Stata.

**Figure 9:**
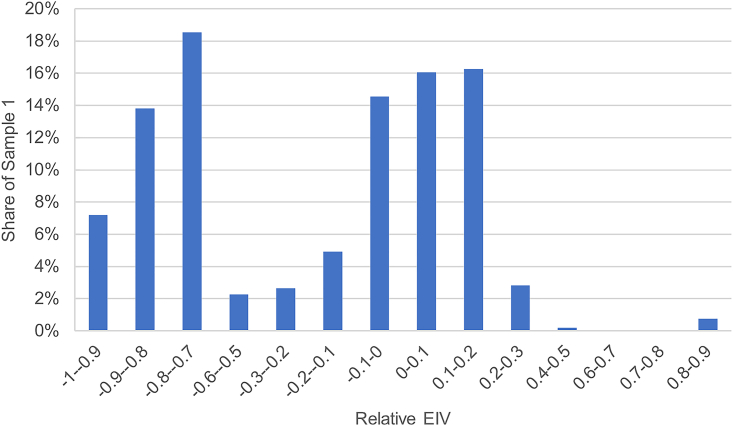
Distribution of relative EIV in sample 1 Counts are shown after removing the observations where borrower-related financial information (e.g., leverage, profitability) was missing, as they would be excluded from the regression in Stata.

A summary of the composition of the two samples can be found in Table 2 (second-hand ships, secured, project finance, and short maturity) and in the supplemental information (deal purpose, repayment type, shipping segment, and shipowner size). Most of the loans in sample 1, and all the loans in sample 2 by construction, concern recourse loans without the use of an SPV. Most of the loans in sample 1 are secured by a collateral, which is in line with Stopford,^67^ which argues that ship finance loans typically are. On the other hand, the minority of the loans in sample 2 are, which suggests that most of this sample is not dedicated to ship finance. The majority of sample 1 concerns newbuilds. This bias might be explained by two reasons. First, they are more easily identified when matching ships to loans (see details on the process in the next section). Second, the dataset might be biased toward newbuilds, as only includes syndicated loans, which are usually used to finance larger transactions. Finally, only a minority of the loans have been provided by Poseidon signatories. This is because the Poseidon Principles were introduced quite late in the period of the sample (2019) and there are more data points before 2015 than after 2015 (see the supplemental information). Figure 8 shows that most of the borrowers in sample 2 have scores ranging from B to D, with few having really poor scores (E), and none reaching the top score (A). Figure 9 shows that sample 1 includes a large range of carbon intensity, with some loans financing ships nearly twice as carbon intensive as their cohort’s average (relative EIV > 0.8), and many ships having a very low-carbon intensity, with EIV 70%–90% lower than their cohort’s average. A slight majority of ships have a carbon intensity close to average (up to 20% more/less carbon intensive than the average), but given the large amount of very efficiency ships in the sample, it is somewhat biased toward more efficient ships, with EIV being on average 30% lower than the ships cohorts'.

**Table 2:** Summary statistics of the dummy variables

Variable	Level	Sample 1	Sample 2	Full dataset
Short maturity	0	498	1,633	7,847
	1	31	3,234	10,899
Project finance	0	468	0	18,461
	1	61	4,867	285
Collateral	0	48	4,340	14,544
	1	481	527	4,202
Second-hand	0	402	N/A	N/A
	1	127	N/A	N/A
SPV	0	483	4,867	18,471
	1	46	0	275
Poseidon Principles	0	512	4,769	18,389
	1	17	98	357

Counts are shown after removing the observations where borrower-related financial information (e.g., leverage, profitability) are missing, as they would be excluded from the regression in Stata.

### Loans-ships matching algorithm

Because it is not publicly known which ships are financed by each loan, the construction of the dependent variables representing the climate performance requires the development of an algorithm to match individual ships to the loans. This algorithm is shown in more detail in this section.

Data on existing and ordered ships were collected on Clarksons’ WFR, and data on loans were taken from Dealscan dataset. There is no direct correspondence, however, between ships listed in Clarksons’ WFR and the loans listed in Dealscan. We developed an algorithm to provide a “best guess” of which ships were financed by specific loans. This algorithm can be broken down in three steps:

(1) First, the correspondence between the list of borrower companies from Dealscan, and shipowners from Clarksons’ WFR, was built.
(2) In parallel, for many loans, the exact ships financed could be identified based on qualitative data given in Dealscan. Using this subset, the average time lag between (1) the active date of the loan and (2) the build date of the ship, was calculated.
(3) Finally, the ships were attached to single loans by matching shipowners/borrowers and build dates/loan-active dates.

**Figure 10:**
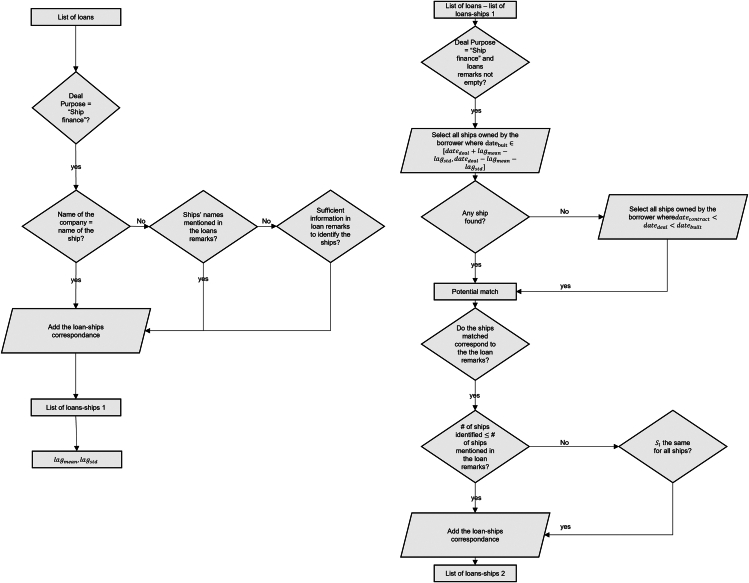
Graphic representation of the loans-ships matching algorithm

Figure 10 shows how steps 2 and 3 were carried out. The following paragraphs describe in more detail the three steps.

#### Matching borrowers to shipowners

In the first step, we identified the correspondence between:

• Borrowers in Dealscan, identified by the website provided (when available), the stock exchange name (when listed and provided), or their name; and
• Shipowners and shipowner groups in Clarksons’ WFR, identified by the website provided (when available), the stock exchange ticker (when listed and provided), or their name.

When an exact correspondence was found between either website, stock exchange name or names (in this specific order), those were automatically matched. For the others, an algorithm was run on the list of names to find the closest possible names, and the results were manually checked to find the correspondences between borrowers/shipowners or borrowers/shipowner groups. Note that this step probably missed some correspondences—when the company had changed names, for example—but there is confidence that the correspondences found were properly matched. The step was very time-consuming and a total of 338 borrowers/shipowners correspondences (with ships, owners, owner groups, or former owner) were identified. Those borrowers were identified as “shipowners,” and the loans that they were awarded constitute the sample of corporate loans. Those shipowners have obtained 808 loans from 2010 to 2021 in our sample, corresponding to $1,053 billion.

#### Manual sample construction and statistics on the loans-to-built lag

Once a ship has been contracted at a shipyard (contract date in the following), shipowners typically need to make pre-delivery payments to the shipyard, which may be covered by a pre-delivery credit, if it has been arranged or by the shipowner’s own funds,^67^ and a post-delivery payment, which is often the largest payments and is made on delivery of the vessel (build date in the following). The latter is typically obtained from commercial bank loans, leasing, or shipyard credit schemes.^67^ If pre- and post-delivery payments are covered by banks loans, it would have been agreed beforehand in a loan agreement.

To explore the timing between the contract date, loan-active date, and the build date, we first manually identify, for a sample of loans, the ships that have been financed. For some loans, a special vehicle was created to act as a borrower for one specific ship. Those loans have been identified because the name of the borrower is the same as the name of the ship or the hull, and the loan purpose is “ship finance.” Furthermore, most of the loans categorized as ship finance contain qualitative information in the columns “deal remark,” “tranche remark,” and “purpose remark.” This information often directly mentions the ships financed, either by their names or by giving characteristics such as builder, ship type, size, build date, and number of ships financed. When this information was sufficient to uniquely identify the ships described, it was matched. The combination of those two methods allowed a manual matching of loans to ships to be built, which is called “list of loans-ships 1” in Figure 10.

Furthermore, this sample was used to identify some characteristics related to the lag between the date at which the loan was active and the date at which the ship was built, which were used in step 3. The average and standard deviation of this lag $lag_{mean}$ and $lag_{std}$ could be computed. One could also use the lag between the ship contract date and the loan-active date. However, it appeared that the loan deal date was closer to the build date than the contract date and that the dispersion of loan-to-deal lag was larger than the contract-to-deal lag. Based on this, it was considered that the lag between loan date and build date was a more robust indicator.

#### Matching ships to loans

Apart from the loans clearly identified, it was not possible to find a direct correspondence between the ships and a loan. For those deals, ships were matched to each of the loans where the ship build date was found to be close enough to the expected build date from the loan data. The date was considered “close enough” when they met one of the following two criteria:

Criterion 1: select all ships of the shipowner where the below two conditions were met:

(Equation 2) $${date}_{buit}\in \left[date_{deal}+lag_{mean}-lag_{std},date_{deal}-lag_{mean}-lag_{std}\right]$$

With $lag_{stdev}$ the standard deviation of the lags of the 12 identified loans.

Criterion 2: select all ships of the shipowner where the debt deal is reached between the date at which the ship is contracted to the shipyard and the date the ship is built:

(Equation 3) $$date_{contract}>date_{loan}>date_{built}$$

With $date_{contract}$ the date at which the ship is contracted to the shipyard, from Clarksons’ WFR.

Before being added to the list of loans-ships 2 (see Figure 10), the results were manually checked against the qualitative data included in the deal remark, tranche remark, and purpose remark. When the ships identified did not correspond to the remarks, when the remarks did not give any information on the ships, or when the loan covered not only ships but also other transactions, the loan was not included. For example, they might be of the wrong segment or built by another shipyard than mentioned. When the ships identified corresponded to the qualitative information contained in the loan’s remarks, but more ships were identified than expected based on those remarks, they were added onto the list of loans-ships 2 only when their characteristics to be included in the regression (size quintile, age, and segment) were identical.

#### Resulting dataset

The matching results have been validated with one shipping lender, who confirmed that the matching was correct for 89% of the ships identified and classified as ship finance. However, the sample of loans reported in Dealscan covers only a small part of the total loan activity (roughly 10% according to the person validating the sample) and is especially scarce after 2019.

The total amount provided to finance ships reported in the full dataset is $69 billion, which is roughly 15% of the total shipping debt over the period (total $440 billion calculated from Petropoulos, 2021.^68^ For 104 of the 224 ship finance deals, no ship could be matched or the loan was not only used for ship finance. For a further 30 deals, EIV data were not available because there was no past observation yet. The remaining sample of observations used for the regression covers $30 billion of debt provided.

### Regression model

Let us now turn to the choice of dependent variables in the model. The dependent variable regressed is the AISD of the loan, i.e., the basis points (bps) over the LIBOR. Note that one unique loan transaction (defined by a unique date-borrower combination) can correspond to several data points if more than one lender is lending and/or various loan characteristics ($L_{l}$) are applied. Typically, a loan can be made in two tranches with two different margins (AISD in Dealscan) and tenors; each tranche is usually financed by more than one lender. The various data points corresponding to a single loan then have the same borrower- and ship-related information. The empirical model used is described by Equation 4 below:

(Equation 4) $$AISD_{lbft}=\alpha_{0}+\alpha_{1}\;{EVI}_{l}+\alpha_{2}\;{EV}_{l}\times Post2015+\alpha_{4}\;L_{bft}+\alpha_{5}\;F_{ft}+\alpha_{6}M_{t}+\alpha_{7}S_{l}+\epsilon_{lbft}$$

with l subscripts indicating a unique loan deal, b the lender, f the borrowing company, and t the time. ${EV}_{l}$ stands for the climate performance and corresponds to the carbon intensity attached to the loan, which is a function of the carbon intensities of the ships financed (EIV) or of the climate rating of the borrower (CDP score). Post2015 is a dummy variable that takes the value 1 after 2015 (date of the Paris Agreement), 0 otherwise. $L_{bft}$, $F_{f}$, and $S_{l}$ are vectors of loan, lender, and ship characteristics that might affect the margin. $M_{t}$ is a variable capturing the state of the newbuilding market. $\alpha_{0}$ is a vector of fixed effects (year, borrower country, and constant). $\epsilon_{lbft}$ is the remaining variation.

There is already an extensive literature on the drivers of loan margins for corporate loans, so that the control variables included in the model used on the corporate finance sample (sample 2) were directly informed by those articles.^20^^,^^22^ Regarding loan characteristics, we control for the loan amount, tranche amount, number of lenders, collateral, repayment type (e.g., revolving loans, term loans), maturity, and a series of dummy variables representing loan purpose (e.g., general purpose, refinance, ship finance). Regarding borrowers’ characteristics, we include company size (total assets), leverage (ratio of debt over assets), and profitability (ratio of return after tax on total assets). Loan characteristics were taken from Dealscan directly; companies-related data are taken from Refinitiv-Eikon. We further control for the state of the ship price market by including the 5-year-old Clarkprice index. This indicator is provided by Clarksons’ shipping intelligence network (SIN). It is calculated as a weighted average of 5-year-old second-hand prices for the largest vessel types (oil tankers, bulk carriers, container, and gas tankers) by the number of vessels in each fleet sector.

There is no econometric literature to the knowledge of the authors on the drivers of loan margins in ship finance. We used the WALS procedure^44^ on an original large list of variables to select a subset of controls. The initial list was compiled by including traditional margin drivers identified in the literature (those used in the regression model used on the corporate finance sample and tranche amount, a dummy for short maturity, a dummy for project finance and whether the borrower is an SPV, and borrower’s capitalization) and additional variables that were suggested by the interviewed financiers (a ship’s second-hand price index to represent market dynamics). We further include the characteristics related to the ships: the average age of the financed ships, the shipping segment of the ships financed, and the size quintile of the financed ships (the quintiles are calculated for each segment). After using the WALS procedure on the full list of variables, we removed from the list of original variables all those whose t ratio is lower than 1 in absolute value, as suggested by De Lucas.^44^ The results of the WALS procedure can be found in the supplemental information. We further add to the model a series of dummy variables on the repayment types, and test for their joint significance by using a Wald test. The Wald test rejects the null hypothesis that the coefficients of those dummy variables are jointly equal to zero, so we include them in the final model. As capitalization and company size are highly correlated, as shown in the correlation matrix in the supplemental information, we further remove capitalization, which barely affects the R-square of the model.

There might be further unobserved heterogeneity in the samples that might alter the results (omitted variables issues). To control for this, we further include in both corporate and ship model specifications fixed effects for years and borrower countries. We test for their joint significance through a Wald test on borrower country and time dummies. The Wald tests reject the null hypothesis at the 1% level for both corporate and ship model specifications that the coefficients of those dummy variables are jointly equal to zero. Therefore, we keep those dummy variables in the final model specification. Furthermore, the errors are likely to be clustered at the company (borrower) level, as argued in Kempa et al.^22^ The coefficients of the model are computed using an OLS regression with robust standard errors clustered at the company level, including time and borrower country dummies.

We use the logarithm of some control variables to improve the readability of the results (see details Table 3). The results of those should therefore be interpreted as follows: an increase in the independent variable by 1% increases the loan margins by $\beta_{i}/100$ bps.

**Table 3:** Description of the regression variables

Name in Equation 2	Variable		Unit	Description	Source
$AISD_{lbft}$	all in spread drawn (AISD)		bps	margin over LIBOR	Dealscan
Climate performance (EV)	carbon disclosure project (CDP) score		N/A	the CDP scores were coded from 0 to 8 with 0 being the lowest (E) and 8 the highest (A)	carbon disclosure project
	Refinitiv		N/A	Refinitiv climate score (0–100)	Eikon-Refinitiv
	relative annual efficiency ratio (AER)		N/A	ship annual efficiency ratio relative to its cohort average AER	Fuel Use And Emissions (FUSE)
	relative estimated index value (EIV)		N/A	ship EIV relative to its cohort average EIV	Fuel Use And Emissions (FUSE)
	energy saving technology		share of number of ships	share of the ships financed that are equipped with at least one energy saving technology, as registered in Clarksons	Clarksons’ WFR
Loan characteristics ($L_{bft})$	loan amount		logarithm of loan amount, in million USD	N/A	Dealscan
	tranche amount		logarithm of tranche amount, in million USD	N/A	Dealscan
	number of lenders		logarithm of number	N/A	Dealscan
	collateral		dummy	dummy equal to 1 if the loan is secured by a collateral	Dealscan
	repayment type		dummy	series of dummy variables corresponding to the type of repayment (e.g., revolving loans, term loans)	Dealscan
	loan purpose		dummy	series of dummy variables corresponding to the purpose of the loan (e.g., general purpose, refinance, ship finance)	Dealscan
	performance		dummy	dummy equal to 1 if the loan includes performance pricing	Dealscan
	Poseidon Principles		dummy	dummy equal to 1 if the lender had signed the Poseidon Principles at the time of the loan	Poseidon Principles website
	maturity		logarithm of the tenor in months	loan tenor	Dealscan
	project finance		dummy	dummy equal to 1 if the loan is used in project finance	Dealscan
	short maturity		dummy	dummy equal to 1 if the tenor of the loan <5 years	Dealscan
Borrower characteristics ($F_{ft}$)	company size		logarithm of loan amount, in USD	logarithm of the borrower’s total assets	Eikon
	leverage		logarithm of ratio	borrowers’ total debt/total assets	Eikon
	SPV		dummy	dummy equal to 1 if the borrower is a special vehicle	Dealscan
	profitability		N/A	borrower net income (after tax profit)/total assets	Eikon
Assets characteristics ($S_{l})$	ship’s size		N/A	average quintile of ship size compared with ship segments	Clarksons’ WFR
	age		years	average age of the ships financed at the time of the loan (0 for newbuilds)	Clarksons’ WFR
	shipping segment		dummy	series of dummies corresponding to the shipping segment (chemical tankers, containers, etc.) of the ships financed	Clarksons’ WFR
Market ($M_{t}$)		second-hand price index	logarithm of index	5-year-old Clarkprice index	Clarksons’ SIN

Furthermore, a summary of the regression variables included in the model is provided in Table 3 and the summary statistics of the independent variables before the logarithm transformation in Table 4.

**Table 4:** Descriptive statistics of the continuous control variables

	Obs.	Mean	SD	Min	Max
All in spread drawn (bps)	18,463	165	110	1	1,250
Carbon disclosure project (CDP) score (E = 0, A = 8)	5,276	5	2	0	7
Refinitiv combined score	10,201	129	31	21	193
Relative annual efficiency ratio (AER)	779	−0.3	0.5	−1.0	2.9
Relative estimated index value (EIV)	779	−0.2	0.4	−1.0	0.9
Energy-saving technology	846	0.3	0.4	N/A	1.0
Loan amount (million USD)	18,744	3,260	4,670	9,98	45,000
Tranche amount (million USD)	18,745	1,510	2,140	2	25,000
Number of lenders	18,705	26	22	1	94
Maturity (months)	18,685	45	32	–	722
Firm size (million USD)	15,400	48,100	54,100	2,809,517	510,000
Leverage	14,774	0.4	0.2	0	1.7
Profitability	15,325	0.0	0.1	−1.1	0.7
Newbuilding price	18,746	134	7	121	162
Age	940	1.0	3.5	0	40.0
Size quintile	830	4.3	0.8	1.0	5.0

Variables are summarized before logarithm transformation.

### Detailed results

The detailed results for the models plotted in the main text are presented in Tables 5, 6, and 7.

**Table 5:** Climate performance pricing and the Paris Agreement, detailed results

	(1) sample2/2010–2021	(2) sample2/2010–2021	(3) sample1/2010–2021	(4) sample1/2010–2021
CDP score	−3.179 (0.587)	0.567 (0.912)	N/A	N/A
Post-2015 dummy = 1 # CDP score	N/A	−35.37∗∗ (0.027)	N/A	N/A
Relative EIV	N/A	N/A	−3.159 (0.930)	−55.42 (0.231)
Post-2015 dummy = 1 # relative EIV	N/A	N/A	N/A	142.5 (0.116)
Loan amount	7.135 (0.493)	4.891 (0.571)	N/A	N/A
Tranche amount	−5.180 (0.390)	−5.382 (0.360)	7.467 (0.239)	6.808 (0.202)
Number of lenders	2.510 (0.847)	1.698 (0.890)	0.0136 (0.999)	−2.204 (0.821)
Maturity	28.36∗∗∗ (0.005)	26.80∗∗∗ (0.007)	−29.27 (0.152)	−33.99 (0.114)
Firm size	−21.21 (0.151)	−18.69 (0.184)	−43.54∗∗∗ (0.001)	−36.87∗∗∗ (0.005)
Leverage	22.00 (0.360)	25.95 (0.261)	37.34 (0.467)	88.61∗ (0.073)
Profitability	80.44 (0.706)	67.61 (0.743)	−120.9 (0.224)	−146.9 (0.132)
Collateral = 1	116.6∗∗ (0.018)	112.6∗∗ (0.017)	33.71∗∗ (0.046)	37.10∗∗∗ (0.002)
Second-hand price index	N/A	N/A	40.21∗∗∗ (0.001)	40.74∗∗∗(0.001)
Ship’s size	N/A	N/A	−17.92 (0.162)	−33.07 (0.125)
Short maturity = 1	N/A	N/A	47.08 (0.224)	36.28 (0.335)
Project finance = 1	N/A	N/A	13.54 (0.274)	13.96 (0.223)
SPV = 1	N/A	N/A	85.15∗∗ (0.015)	85.92∗∗ (0.023)
Year FE	yes	Yes	yes	yes
Borrower country FE	yes	yes	yes	yes
Repayment type	yes	yes	yes	yes
Shipping segment	no	no	yes	yes
Industry FE	no	no	no	no
Borrower FE	no	no	no	no
R-squared	0.804	0.812	0.912	0.919
Observations	4,867	4,867	463	463
BIC	53,654.0	53,461.4	4,309.0	4,274.7
AIC	53,400.9	53,201.8	4,184.9	4,150.6

*p* values in parentheses. FEs, fixed effects; BIC, Bayesian information criterion; AIC, Akaike information criterion.

∗ *p* < 0.10.

∗∗ *p* < 0.05.

∗∗∗ *p* < 0.01.

**Table 6:** Climate performance (period breakdown) pricing before and after the Paris Agreement, detailed results

	(7) sample2_2010–2015	(8) sample2_2015–2021
CDP score	2.626 (0.678)	-46.15∗∗∗ (0.005)
Relative EIV	N/A	N/A
Loan amount	−5.846(0.468)	51.87∗∗ (0.028)
Tranche amount	−6.448 (0.245)	−12.64 (0.460)
Number of lenders	−3.743 (0.706)	50.35 (0.180)
Maturity	20.73∗∗ (0.027)	15.66 (0.503)
Firm size	−17.44 (0.354)	−34.07∗∗(0.020)
Leverage	32.39 (0.190)	−15.92 (0.659)
Profitability	310.3 (0.143)	−445.4∗∗∗ (0.009)
Collateral = 1	10.66 (0.541)	221.9∗∗∗ (0.000)
Second-hand price index	N/A	N/A
Ship’s size	N/A	N/A
Short maturity = 1	N/A	N/A
Project finance = 1	N/A	N/A
SPV = 1	N/A	N/A
Year FE	yes	yes
Borrower country FE	yes	yes
Repayment type	yes	yes
Shipping segment	no	no
Industry FE	no	no
Borrower FE	no	no
R-squared	0.875	0.928
Observations	4,097	770
BIC	42,445.4	8,354.1
AIC	42,243.2	8,242.6

*p* values in parentheses. FEs, fixed effects; BIC, Bayesian information criterion; AIC, Akaike information criterion.

∗ *p* < 0.10.

∗∗ *p* < 0.05.

∗∗∗ *p* < 0.01.

**Table 7:** Poseidon Principles and climate pricings, detailed results

	(5) sample2/2010-2021	(6) sample1/2010-2021
CDP score	−2.367 (0.676)	N/A
Poseidon Principles signatory = 1 # CDP score	−56.02∗∗∗ (0.001)	N?A
Relative EIV	N/A	−13.59 (0.718)
Poseidon Principles signatory=1 # relative EIV	N/A	55.29 (0.126)
Loan amount	5.255 (0.588)	N/A
Tranche amount	−5.597 (0.341)	7.277 (0.235)
Number of lenders	3.446 (0.787)	0.116 (0.990)
Maturity	28.60∗∗∗ (0.004)	−30.14 (0.140)
Firm size	−19.36 (0.171)	−42.03∗∗∗ (0.001)
Leverage	22.94 (0.338)	47.63 (0.331)
Profitability	85.58 (0.683)	−127.7 (0.193)
Collateral=1	114.5∗∗ (0.020)	33.87∗∗(0.031)
Second-hand price index	N/A	39.53∗∗∗ (0.001)
Ships' size	N/A	−21.61 (0.122)
Short maturity=1	N/A	45.47 (0.237)
Project finance=1	N/A	14.00 (0.243)
SPV=1	N/A	80.20∗∗ (0.025)
Year FE	yes	yes
Borrower country FE	yes	yes
Repayment type	yes	yes
Shipping segment	no	yes
Industry FE	no	no
Borrower FE	no	no
R-squared	0.809	0.914
Observations	4,867	463
BIC	53,561.7	4,301.5
AIC	53,295.6	4,177.4

The model was not run on the ship finance sample, as it was too small and showed signs of over fitness.

*p* values in parentheses. FEs, fixed effects; BIC, Bayesian information criterion; AIC, Akaike information c.riterion

∗ *p* < 0.10.

∗∗ *p*< 0.05.

∗∗∗ *p*< 0.01.

**Table 8:** List of interviews

Name	Type	Location	Poseidon Principles	Size
Interview 1	Commercial bank	North America	yes	$5–10 billion
Interview 2	Commercial bank	Western Europe	yes	>$10 billion
Interview 3	Alternative lender	Western Europe	no	$0–5 billion
Interview 4	Commercial bank	Western Europe	yes	>$10 billion
Interview 5	Commercial bank	Western Europe	yes	>$10 billion
Interview 7	Commercial bank	Asia	yes	>$10 billion
Interview 9	Commercial bank	Western Europe	yes	$5–10 billion
Interview 11	Commercial bank	Asian branch of a North American bank	yes	$5–10 billion
Interview 13	Commercial bank	Western Europe	yes	$5–10 billion

### Interview data collection

This article draws on data from 9 in-depth interviews with financiers covering around 27% of the shipping debt, conducted between May and November 2022, to validate the quantitative results and investigate their drivers. 8 interviews have been conducted with commercial banks active in shipping and 1 with an alternative lender specialized in shipping decarbonization (Table 8). All were mostly providing shipping debt to the industry, although some would also provide a range of products in addition to debt. All interviews were conducted with senior managers of financial companies involved in the shipping segment. One interview was conducted face-to-face and the others virtually. Interviews typically lasted an hour. All interviews were recorded. Interviews were guided along a general interview guide but were left semi-structured.

## Supplemental information

Document S1. Supplemental experimental procedures, Figures S1–S3, and Tables S1–S14

Document S2. Article plus supplemental information
