# Supplementary material for: Lower margins are tied to companies’ climate performance rather than to low-carbon assets
Source: Cell Rep Sustain. 2024 Aug 23;1(8):100155. doi: 10.1016/j.crsus.2024.100155 (PMC11378609; doi:10.1016/j.crsus.2024.100155)
Supplement: Document S1. Supplemental experimental procedures, Figures S1–S3, and Tables S1–S14 [file mmc1.pdf]

CRSUS, Volume *1*

Supplemental information

Lower margins are tied to companies' climate performance rather than to low-carbon assets

Marie Fricaudet, Sophia Parker, Nadia Ameli, and Tristan Smith

Supplemental Experimental Procedures

Choice of regression variables

WALS estimates - Weibull			
	Number of observations	=	492
	k1	=	2
	k2	=	14
	q	=	0.8876
	alpha	=	0.1124
	c	=	0.6931
	sigma	=	52.1235

all_in_spread_drawn_bps	Coef.	Std. Err.	t	[1-Std. Err. Bands]	
_cons	494.3	122.1	4.1	372.2	616.4
Relative EIV	-43.3	8.9	-4.9	-52.1	-34.4
Deal Amount	2.3	5.1	0.5	-2.8	7.4
Tranche amount	9.6	3.4	2.8	6.2	13.1
Number of lenders	-33.6	5.6	-6.0	-39.2	-28.0
Maturity	-20.7	8.7	-2.4	-29.3	-12.0
Firm size	-22.9	3.6	-6.4	-26.5	-19.4
Leverage	55.6	12.5	4.5	43.1	68.1
Profitability	49.3	43.7	1.1	5.7	93.0
Second-hand price index	1.7	0.9	1.9	0.8	2.6
Age	-0.4	1.5	-0.3	-1.9	1.1
Ships' size	27.9	5.2	5.3	22.6	33.1
Collateral dummy	23.2	15.2	1.5	8.0	38.4
Short maturity	18.1	9.1	2.0	9.1	27.2
Project finance	21.9	14.6	1.5	7.3	36.4
SPV	494.3	122.1	4.1	372.2	616.4

Table S1: Results of the WALS procedure on the ship sample

Sample bias

Table S2 shows the control variables used in our models in the various samples of estimation. On average, the loans provided are much larger in the corporate finance sample than when only financing ships. The average number of lenders is also lower in the ship samples than in the corporate finance samples. Not surprisingly, the large majority of the loans in the ships sample are secured by a collateral, which is likely to be secured by the financed ship directly. On the other hand, only a minority of corporate loans are secured.

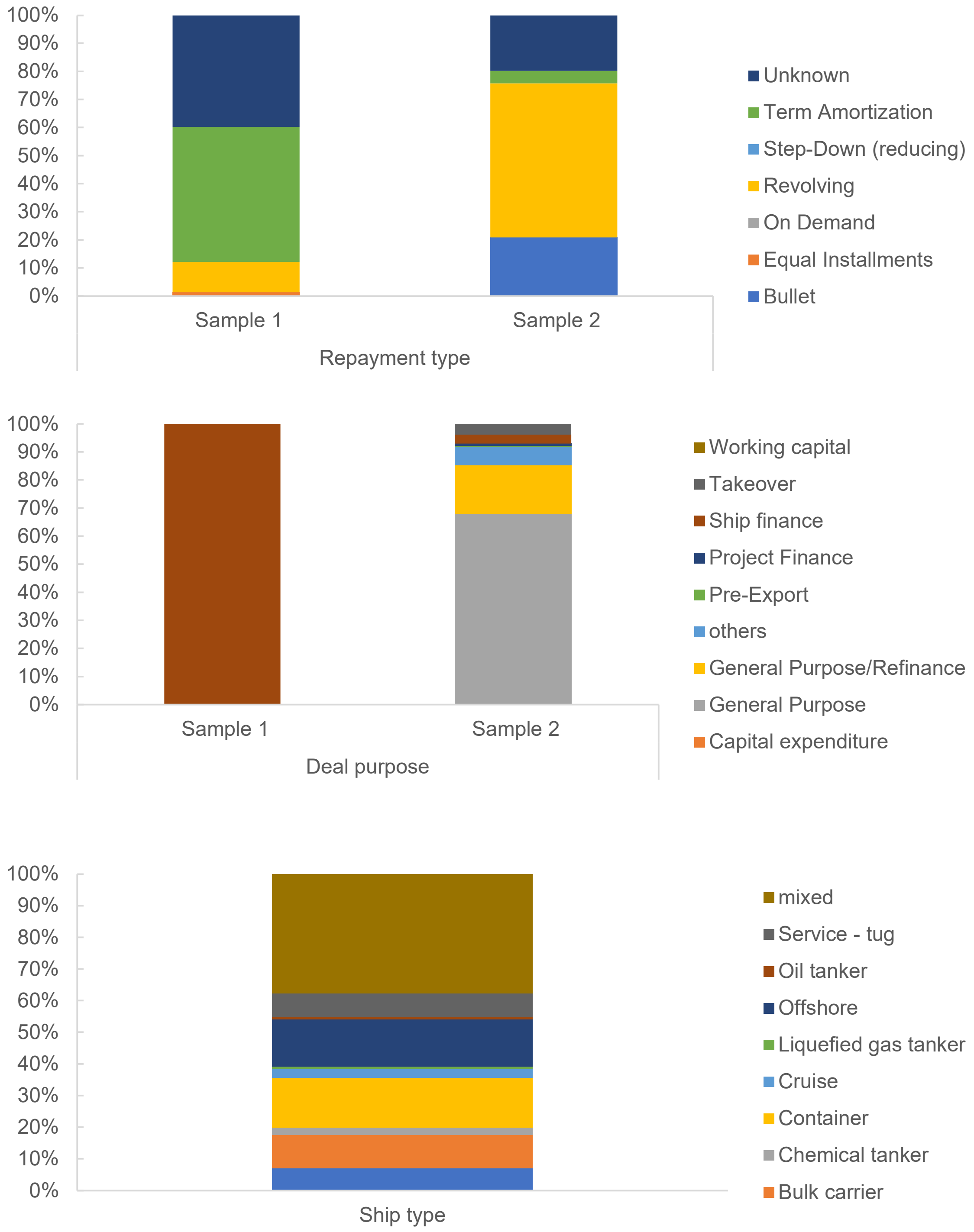

Figure S1: Samples composition

The labels correspond to the number of observations

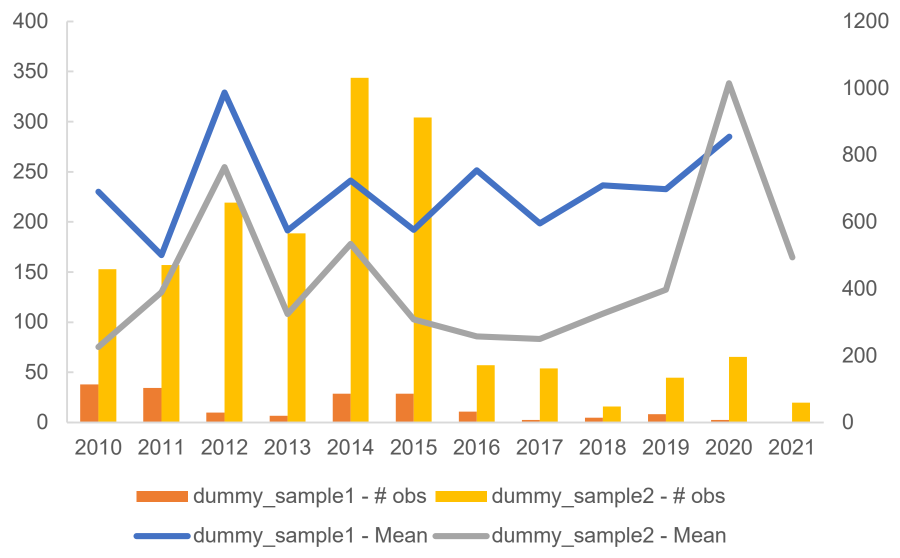

Figure S2: Observations per year

To check whether those samples are biased, we compare the average leverage, profitability and company size to all companies classified under the NAIC “Deep sea, coastal and Great Lakes water transportation”, and to all companies which provided a ticker in Clarksons over the period 2010 to 2021. The results can be found in Figure S3. It is first worth noting that even those two samples might be biased compared to the average shipowner, as most shipowners do not report publicly that information. Second, those two samples do not compare well with each other, with companies classified under the NAIC “Deep sea, coastal and Great Lakes water transportation” being significantly small than those who reported in Clarksons which have a ticker. It can be expected that only the largest shipowners would report publicly their information and/or be publicly listed.

Borrowers of our corporate finance sample (sample 2) are much larger than the average company classified under the NAIC “Deep sea, coastal and Great Lakes water transportation” and larger than the average Clarksons company reporting a ticker. Borrowers who borrowed money to finance ships (samples 1) are on average much smaller and are a bit more leveraged than those of the corporate finance sample and of shipowners who have displayed a ticker. They are however larger than the average firm of NAIC “Deep sea, coastal and Great Lakes water transportation” and have similar leverage. This suggests that both samples, but in particular the corporate sample, are biased towards large firms in terms of assets.

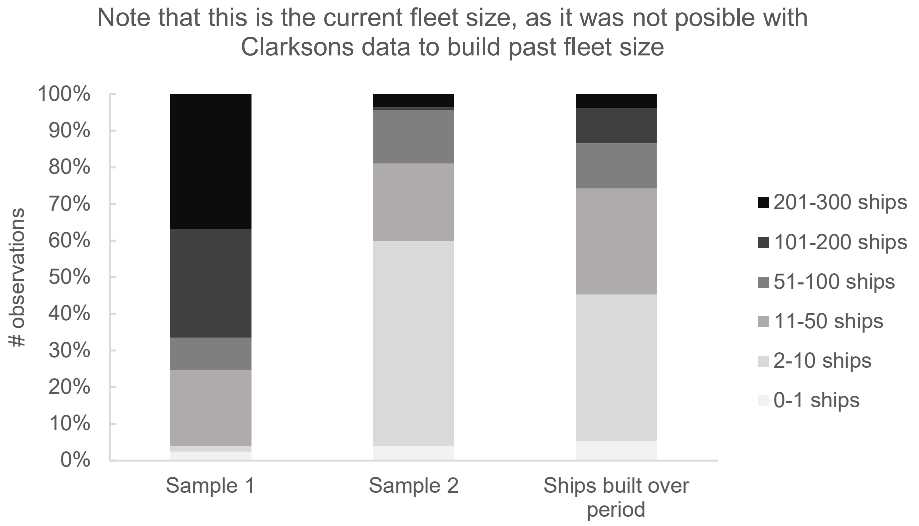

Figure S3: Shipowner size by sample

a. *The first four columns represent the number of observations in the sample.*
b. *The last column corresponds to the number of ships built between 2010 and 2021.*
c. *The numbers plotted correspond to current fleet size of the shipowner rather than fleet size at the time of loan provision, as it was not possible from Clarksons data to build past fleet size by shipowner*

d.	**Sample 1**	**Sample 2**	**Selected NAIC**	**Clarksons owners with ticker**
AIDS (bps)	220	150		
Number of Lenders	9	31		
Loan Amount (million USD)	650	5,292		
Tranche Amount (million USD)	325	2,502		
Maturity (months)	88	41		
Firm Size (million USD)	9,436	68,770	2,299	54,355
Profitability	0.02	0.01	0.00	0.01
Leverage	0.48	0.35	0.43	0.34
CDP score (E=0, A=8)	2.3	4.8		
Relative EIV	- 0.32			

Table S2: Average of continuous control variables in Sample 1 and 2

The variables are summarized before the logarithm transformations

We further check the bias of the sample by comparing the average fleet size of the shipowner, expressed in number of ships, compared to the average fleet size in Clarksons. Figure S3 shows the results. It is worth noting that the fleet size corresponds to the current fleet size, not the fleet size at the time when the loan was emitted. This is because the Clarksons WFR does not provide exhaustive

information on the ships which have been scrapped or sold in the past, especially in the beginning of the sample period, so the past fleet size could not be computed.

The figure clearly shows that our ship sample (sample 1) biased towards larger shipowners compared to the total sample of shipowners. This could have two main explanations. First, it is likely that larger shipowners have a disproportionately large access to the debt market to finance ships. This was confirmed during the interviews. So although our samples are not representative of the shipowners in general, they might not be biased compared to the average shipping loan. A second explanation is that our samples are biased compared to shipping loans in general, as they only cover a small part of the total debt provided to shipowners. This might be the case because Dealscan only contains syndicated loans, which are often used to finance larger amount and might therefore only be available to larger shipowners. On the other hand, our corporate sample is not particularly biased against small shipowners – actually it is slightly biased towards them.

Robustness analysis

To check the robustness of the results, we conducted several robustness checks. The results are in the supplementary materials.

We first recalculated the model using alternative measures of the corporate environment rating (Refinitiv environmental score, which equals to the Refinitiv environmental score – controversies score) and ship environmental rating (AER, energy saving technology installed) and by removing ship finance from the corporate sample (sample 4). Furthermore, to address potential influences on our primary findings, we conducted additional analyses by re-estimating equation (2) in the main text employing alternative sets of dummy variables. First, we incorporated dummy variables to account for the specific industry within which the companies operate, as classified under the "major industry group" as provided by Dealscan. Finally, to incorporate unobservable company characteristics that remain constant over time, we incorporated company dummies into both models during estimation. By doing so, the derived coefficients can be interpreted as specific effects within each company.

This latter sensitivity check could only be conducted on the corporate samples, as the ships' sample was too small and showed signs of overfitness.

A summary of the robustness analysis is provided in Table S3**Error! Reference source not found.**. From this, it appears that the positive pricing of corporate climate performance after the Paris Agreement is robust across most model specifications when using CDP as a measure of climate performance (although the significance is lower in some model specifications). The results using Refinitiv environmental score points to the same direction, but the results are inconsistent between various model specifications, suggesting that the Refinitiv environmental score is not a robust variable. Similarly, the positive pricing of climate performance at the corporate level by Poseidon signatories is robust across model specifications (samples, industry and borrowers fixed effects) when measured by the CDP performance. The results are not robust however not using when using the Refinitiv environmental score, with the coefficients becoming insignificant. This might be explained by the fact that the Refinitiv environmental score is more recent, less trusted and less used by investors [44]. Furthermore, it covers a larger range of and is therefore less presentative of climate risk. Its correlation with the CDP score is low (both coefficients are 17% correlated in our sample; Refinitiv environmental score is 55% on average correlated with major ESG environmental score metrics across all industries [29]). This discrepancy in results and lack of correlation highlights the lack of adequate tools to measure the climate performance of enteprises. Pricing of corporate climate performance before 2015 is never found to be significantly significant when using the CDP score, but is found to be significantly positive in several model specifications when using the Refinitiv environmental score, so that the results are unconclusive.

A robust result across model specifications is that banks have not positively priced ships' carbon intensity before and after the Paris Agreement. It one model specification, the coefficient of relative AER and of relative EIV becomes significant, suggesting that there is negative pricing at least before the Paris Agreement, with more carbon intensive ships enjoying lower margins. However, this result is not robust across model specifications and across proxies for carbon intensity. Finally, there is little evidence that the Poseidon Principles has impacted the pricing of ships'

carbon intensity, as only one of the model specifications shows a significant result, but the level of significant is small (9.6%) and the result is not robust across other model specifications.

Climate risk	Metrics	Period breakdown	Sample	Industry dummies	Firm dummies	Pricing of climate risk whole period	Pricing of climate risk before 2015	Pricing of climate risk after 2015	Poseidon Principles increase the pricing of climate risk
Asset	AER	N	1	N	N				
Asset	AER	Y	1	N	N	NA		NA	NA
Asset	AER	N	1	Y	N				
Asset	AER	Y	1	Y	N	NA	NA	NA	NA
Asset	EST	N	1	N	N				
Asset	EST	Y	1	N	N	NA		NA	NA
Asset	EST	N	1	Y	N				
Asset	EST	Y	1	Y	N	NA		NA	NA
Asset	EIV	N	1	N	N				
Asset	EIV	Y	1	N	N	NA		NA	NA
Asset	EIV	N	1	Y	N				
Asset	EIV	Y	1	Y	N	NA	NA	NA	NA
Corporate	CDP	N	2	N	N				
Corporate	CDP	Y	2	N	N	NA			NA
Corporate	CDP	N	2	Y	N				
Corporate	CDP	Y	2	Y	N	NA			NA
Corporate	CDP	N	2	N	Y				
Corporate	CDP	Y	2	N	Y	NA			
Corporate	CDP	N	3	N	N				
Corporate	CDP	Y	3	N	N	NA			NA
Corporate	CDP	N	3	Y	N				
Corporate	CDP	Y	3	Y	N	NA			NA
Corporate	CDP	N	3	N	Y				
Corporate	CDP	Y	3	N	Y	NA			
Corporate	Refinitiv	N	2	N	N				
Corporate	Refinitiv	Y	2	N	N	NA			NA
Corporate	Refinitiv	N	2	Y	N				
Corporate	Refinitiv	Y	2	Y	N	NA			NA

Climate risk	Metrics	Period breakdown	Sample	Industry dummies	Firm dummies	Pricing of climate risk whole period	Pricing of climate risk before 2015	Pricing of climate risk after 2015	Poseidon Principles increase the pricing of climate risk
Corporate	Refinitiv	N	2	N	Y				
Corporate	Refinitiv	Y	2	N	Y	NA			
Corporate	Refinitiv	N	3	N	N				
Corporate	Refinitiv	Y	3	N	N	NA			NA
Corporate	Refinitiv	N	3	Y	N				
Corporate	Refinitiv	Y	3	Y	N	NA			NA
Corporate	Refinitiv	N	3	N	Y				
Corporate	Refinitiv	Y	3	N	Y	NA			

NA not measured/sample too small
non-significant coefficient
Positive 10% significant
Positive 5% significant
Positive 1% significant
Negative 10% significant
Negative 5% significant
Negative 1% significant

Table S3: Summary of the robustness analysis

a. *Positive corresponds to a positive pricing of climate performance, i.e. a negative coefficient on the CDP/Refinitiv scores (a higher score leads to a lower margin) and a positive coefficient on the AER (a higher carbon intensity leads to a higher margin). Inversely, negative corresponds to a negative pricing of climate performance.*

b. *The colours correspond to the level of significance of the coefficient of interest.*

	Deal amoun t	Tranch e amount	Numbe r of lenders	Collatera l	Short maturit y	Maturit y	Fir m size	Capitalisatio n	Leverag e	Profitabilit y	Second -hand	SP V	Age	Size quintil e	Second -hand

Deal amount	1.00														
Tranche amount	0.71	1.00													
Number of lenders	0.59	0.36	1.00												
Collateral	0.11	-0.05	0.27	1.00											
Short maturity	-0.30	-0.20	-0.18	0.05	1.00										
Maturity	0.13	0.24	-0.26	-0.27	-0.59	1.00									
Firm size	0.56	0.30	0.48	-0.06	-0.07	-0.11	1.00								
Capitalisation	0.54	0.27	0.46	-0.05	-0.06	-0.12	1.00	1.00							
Leverage	-0.21	-0.21	-0.06	0.03	0.05	-0.29	-0.24	-0.23	1.00						
Profitability	0.05	0.12	0.02	0.05	0.00	-0.07	-0.16	-0.19	0.23	1.00					
Second-hand price index	0.02	-0.02	0.24	0.09	-0.20	0.03	-0.06	-0.07	-0.18	0.12	1.00				
SPV	-0.60	-0.47	-0.25	-0.15	0.10	-0.03	-0.16	-0.15	0.24	-0.01	0.02	1.00			
Age	-0.14	0.00	-0.10	0.10	0.23	-0.23	-0.30	-0.32	0.30	0.31	-0.25	-0.09	1.00		
Size quintile	0.48	0.37	0.50	-0.10	-0.14	-0.13	0.44	0.43	-0.02	0.07	0.00	-0.46	0.07	1.00	
Second-hand price index	-0.07	0.09	-0.12	0.16	0.21	-0.28	-0.26	-0.24	0.33	-0.05	-0.32	-0.14	0.53	-0.06	1.00

Table S4: Correlation matrix between the control variables

Interview Guide

Investments decisions – descriptive

1. What types of financial products do you provide to shipowners?
2. How long is the tenor and the profile typically?
3. What type of ships and clients do you finance?
4. If you had to give 3 main factors you consider when deciding whether or not you will provide finance for a ship, which ones would they be?
5. If you had to give 3 main factors which influence the interest rate you give, what would they be?
6. Why signing the Poseidon Principles?

Evolution of the industry over the last decade

7. Could you tell me the story of the first ship investment that you made in your carrier?
8. Could you tell me the story of the last ship investment that you made?
9. Have you observed any evolution in the way your company views and mitigates for climate risks since you joined?

Expectations concerning future stranded assets

10. How do you feel the demand for cargo shipping such as oil, coal and natural gas will evolve in the coming [*insert tenor] years?
11. How likely do you feel it is that it will impact the value of the fleet you finance?
12. How do you feel the pressures to limit carbon emissions from shipping will evolve in the coming [*insert tenor] years?
13. How likely do you feel it is that the ships you finance lose their value because of efforts to limit carbon emissions from shipping?
14. How do you mitigate for those risks (if at all)?
15. Under which conditions would you finance alternative-fuelled ships?

Table S5: Detailed results by period. Central model

	(1)	(2)	(3)	(4)	(5)	(6)	(7)	(8)	(9)	(10)	(11)	(12)	(13)	(14)
	sample2 2010-2015	sample2 2015-2021	sample2 2010-2015	sample2 2015-2021	sample3 2010-2015	sample3 2015-2021	sample3 2010-2015	sample3 2015-2021	sample1 2010-2015	sample1 2015-2021	sample1 2010-2015	sample1 2015-2021	sample1 2010-2015	sample1 2015-2021
CDP score	2.626	-46.15***			0.179	-37.72**								
	(0.678)	(0.005)			(0.978)	(0.031)								
Refinitiv environmental score			-0.736**	0.722*			-0.642*	0.411						
			(0.034)	(0.078)			(0.100)	(0.433)						
Relative EIV									-146.7***	-3842.3***				
									(0.001)	(0.000)				
AER											-138.3***	-123.0***		
											(0.000)	(0.000)		
Energy saving technologies													-42.78	-154.4***
													(0.314)	(0.001)
Loan amount	-5.846	51.87**	-7.565	7.399	-7.979	84.38***	-7.416	0.514						
	(0.468)	(0.028)	(0.298)	(0.531)	(0.368)	(0.001)	(0.337)	(0.972)						
Tranche amount	-6.448	-12.64	-2.354	-6.099	-3.885	-35.86**	-1.938	0.105	6.136	0.0000277	2.618	7.16e-08	5.875	1.476
	(0.245)	(0.460)	(0.561)	(0.464)	(0.559)	(0.029)	(0.669)	(0.988)	(0.152)	(0.309)	(0.358)	(0.316)	(0.440)	(0.484)
Number of lenders	-3.743	50.35	-6.424	36.23**	-3.644	47.09	-6.769	40.05*	1.349	-520.1***	0.469	-520.1***	-0.280	-437.4***
	(0.706)	(0.180)	(0.528)	(0.040)	(0.720)	(0.241)	(0.511)	(0.069)	(0.827)	(0.000)	(0.921)	(0.000)	(0.971)	(0.000)
Maturity	20.73**	15.66	12.60*	8.969	18.33**	31.14	10.58	6.446	-20.75	-0.000326	-22.38	-0.0000125	25.64	-37.50***
	(0.027)	(0.503)	(0.097)	(0.342)	(0.049)	(0.269)	(0.162)	(0.562)	(0.107)	(0.432)	(0.139)	(0.432)	(0.338)	(0.000)
Firm size	-17.44	-34.07**	-2.477	-48.67***	-15.05	-37.72**	-4.488	-48.43***	-33.56***	-58017.6***	-25.72**	2671.8***	-44.98***	-1382.7***
	(0.354)	(0.020)	(0.750)	(0.000)	(0.493)	(0.028)	(0.609)	(0.000)	(0.001)	(0.000)	(0.018)	(0.000)	(0.001)	(0.000)
Leverage	32.39	-15.92	41.65***	28.74	39.69	-11.29	40.77***	13.41	138.6***	149918.0***	164.3***	-7172.1***	61.38	3297.6***
	(0.190)	(0.659)	(0.007)	(0.333)	(0.157)	(0.764)	(0.009)	(0.686)	(0.001)	(0.000)	(0.001)	(0.000)	(0.287)	(0.000)
Profitability	310.3	-445.4***	16.55	-580.3***	392.3*	-430.8**	8.940	-507.7**	-232.9***	4249010.9***	-219.5***	-202676.2***	-224.3**	70730.9***
	(0.143)	(0.009)	(0.876)	(0.005)	(0.063)	(0.016)	(0.949)	(0.014)	(0.007)	(0.000)	(0.010)	(0.000)	(0.010)	(0.000)
Collateral=1	10.66	221.9***	18.90	161.5***	4.747	197.6***	17.09	196.9***	3.040	-180757.9***	25.10	8949.9***	14.35	-3866.7***
	(0.541)	(0.000)	(0.241)	(0.001)	(0.761)	(0.000)	(0.288)	(0.000)	(0.841)	(0.000)	(0.132)	(0.000)	(0.535)	(0.000)
Second-hand price index									13.97	-249.5***	19.34	-102.7***	4.650	-90.96***
									(0.326)	(0.000)	(0.107)	(0.000)	(0.721)	(0.000)
Ships' size									-55.64***	-329.8***	-65.96***	-19.12***	-29.96	31.25***
									(0.001)	(0.000)	(0.001)	(0.000)	(0.105)	(0.004)

	(1) sample2 2010-2015	(2) sample2 2015-2021	(3) sample2 2010-2015	(4) sample2 2015-2021	(5) sample3 2010-2015	(6) sample3 2015-2021	(7) sample3 2010-2015	(8) sample3 2015-2021	(9) sample1 2010-2015	(10) sample1 2015-2021	(11) sample1 2010-2015	(12) sample1 2015-2021	(13) sample1 2010-2015	(14) sample1 2015-2021
Short maturity=1									-29.99	-0.0000254	-40.22	-0.00000174	30.79	-5.380***
									(0.452)	(0.432)	(0.352)	(0.432)	(0.433)	(0.000)
Project finance=1									28.36***		33.75***		30.93***	
									(0.006)		(0.003)		(0.001)	
SPV=1									-38.79		-51.18		-47.00	
									(0.403)		(0.267)		(0.332)	
Year FE	Yes	Yes	Yes	Yes	Yes	Yes	Yes	Yes	Yes	Yes	Yes	Yes	Yes	Yes
Borrower Country FE	Yes	Yes	Yes	Yes	Yes	Yes	Yes	Yes						
Repayment type	Yes	Yes	Yes	Yes	Yes	Yes	Yes	Yes	Yes	Yes	Yes	Yes	Yes	Yes
Shipping segment	No	No	No	No	No	No	No	No	Yes	Yes	Yes	Yes	Yes	Yes
Industry FE	No	No	No	No	No	No	No	No	No	No	No	No	No	No
Borrower FE	No	No	No	No	No	No	No	No	No	No	No	No	No	No
R-squared	0.875	0.928	0.759	0.817	0.887	0.933	0.771	0.832	0.964	1.000	0.964	1	0.934	0.994
Observations	4097	770	7606	2349	3945	738	7255	2132	384	79	384	79	408	103
BIC	42445.4	8354.1	81186.3	25946.9	40516.1	7985.8	77159.5	23468.7	3259.3	-1513.4	3248.7	.	3751.2	636.8
AIC	42243.2	8242.6	80915.8	25745.2	40321.5	7879.9	76904.6	23276.1	3168.4	-1525.2	3157.8	.	3638.9	628.9

p-values in parentheses
* p<0.10, ** p<0.05, *** p<0.01

Table S6: Full results by period - sensitivity - industry dummies

	(1) sample2 2010-2015	(2) sample2 2015-2021	(3) sample2 2010-2015	(4) sample2 2015-2021	(5) sample3 2010-2015	(6) sample3 2015-2021	(7) sample3 2010-2015	(8) sample3 2015-2021	(9) sample1 2010-2015	(10) sample1 2015-2021	(11) sample1 2010-2015	(12) sample1 2015-2021	(13) sample1 2010-2015	(14) sample1 2015-2021
CDP score	5.225	-13.21			3.676	-3.198								
	(0.232)	(0.617)			(0.427)	(0.910)								
Refinitiv environmental score			-1.047***	-0.336			-1.009***	-0.987						
			(0.004)	(0.520)			(0.007)	(0.200)						
Relative EIV									202.3**	-3842.3***				
									(0.011)	(0.000)				
AER											252.4***	-123.0***		
											(0.000)	(0.000)		
Energy saving technologies													-21.70	-154.4***
													(0.677)	(0.001)
Loan amount	-6.450	136.7***	6.567	17.49	-5.746	172.0***	5.757	14.99						

	(1)	(2)	(3)	(4)	(5)	(6)	(7)	(8)	(9)	(10)	(11)	(12)	(13)	(14)
	sample2	sample2	sample2	sample2	sample3	sample3	sample3	sample3	sample1	sample1	sample1	sample1	sample1	sample1
	2010-2015	2015-2021	2010-2015	2015-2021	2010-2015	2015-2021	2010-2015	2015-2021	2010-2015	2015-2021	2010-2015	2015-2021	2010-2015	2015-2021
	(0.364)	(0.000)	(0.328)	(0.184)	(0.425)	(0.000)	(0.431)	(0.370)						
Tranche amount	-0.470	-28.86**	-0.844	-8.608	-1.693	-45.25***	-0.928	-6.091	-1.713	0.0000275	2.172*	7.33e-08	4.933	1.476
	(0.949)	(0.047)	(0.847)	(0.272)	(0.827)	(0.001)	(0.852)	(0.418)	(0.256)	(0.309)	(0.063)	(0.316)	(0.431)	(0.484)
Number of lenders	-6.511	61.41	-14.15	25.19	-4.946	75.20	-14.35	34.21	1.893	-520.1***	3.594	-520.1***	-2.043	-437.4***
	(0.536)	(0.158)	(0.122)	(0.172)	(0.661)	(0.127)	(0.134)	(0.123)	(0.733)	(0.000)	(0.279)	(0.000)	(0.769)	(0.000)
Maturity	22.74**	49.18*	12.36*	16.71*	21.60*	88.87**	11.02	12.26	-15.80	-0.000322	-7.956	-0.0000128	24.33	-37.50***
	(0.044)	(0.071)	(0.092)	(0.089)	(0.055)	(0.022)	(0.129)	(0.249)	(0.127)	(0.432)	(0.330)	(0.432)	(0.352)	(0.000)
Firm size	-15.38	-57.07**	-7.196	-54.85***	-10.46	-73.00***	-5.673	-59.57***	15.62**	-58017.6***	14.83***	2671.8***	-34.35*	-1382.7***
	(0.249)	(0.014)	(0.323)	(0.000)	(0.472)	(0.003)	(0.454)	(0.000)	(0.027)	(0.000)	(0.000)	(0.000)	(0.066)	(0.000)
Leverage	16.15	154.4	34.91**	10.81	14.92	111.6	34.47**	-2.606	17.01	149918.0***	-77.01***	-7172.1***	37.56	3297.6***
	(0.551)	(0.135)	(0.015)	(0.618)	(0.626)	(0.391)	(0.020)	(0.905)	(0.623)	(0.000)	(0.002)	(0.000)	(0.568)	(0.000)
Profitability	-178.6	118.2	46.88	-561.5***	-244.1	336.9	57.35	-536.9***	416.8***	4249011.1***	564.8***	-202676.2***	-117.8	70730.9***
	(0.292)	(0.553)	(0.615)	(0.004)	(0.177)	(0.229)	(0.629)	(0.005)	(0.000)	(0.000)	(0.000)	(0.000)	(0.483)	(0.000)
Collateral=1	27.29*	56.76	31.70**	141.1***	26.40*	-11.66	29.25**	162.4***	115.1***	-180757.9***	90.01***	8949.9***	14.70	-3866.7***
	(0.067)	(0.164)	(0.017)	(0.001)	(0.070)	(0.807)	(0.016)	(0.001)	(0.000)	(0.000)	(0.000)	(0.000)	(0.635)	(0.000)
Second-hand price index									77.04***	-249.5***	72.90***	-102.7***	5.094	-90.96***
									(0.000)	(0.000)	(0.000)	(0.000)	(0.737)	(0.000)
Ships' size									20.45	-329.8***	64.48***	-19.12***	-5.050	31.25***
									(0.166)	(0.000)	(0.000)	(0.000)	(0.882)	(0.004)
Short maturity=1									-4.455	-0.0000250	15.97	-0.00000178	24.71	-5.380***
									(0.875)	(0.432)	(0.360)	(0.432)	(0.487)	(0.000)
Project finance=1									29.42***		19.17***		28.07***	
									(0.000)		(0.000)		(0.000)	
SPV=1									60.67*		115.7***		-11.30	
									(0.057)		(0.000)		(0.856)	
Year FE	Yes	Yes	Yes	Yes	Yes	Yes	Yes	Yes	Yes	Yes	Yes	Yes	Yes	Yes
Borrower Country FE	Yes	Yes	Yes	Yes	Yes	Yes	Yes	Yes						
Repayment type	Yes	Yes	Yes	Yes	Yes	Yes	Yes	Yes	Yes	Yes	Yes	Yes	Yes	Yes
Shipping segment	No	No	No	No	No	No	No	No	Yes	Yes	Yes	Yes	Yes	Yes
Industry FE	Yes	Yes	Yes	Yes	Yes	Yes	Yes	Yes	Yes	Yes	Yes	Yes	Yes	Yes
Borrower FE	No	No	No	No	No	No	No	No	No	No	No	No	No	No
R-squared	0.920	0.959	0.800	0.863	0.928	0.966	0.816	0.887	0.988	1.000	0.992	1	0.940	0.994

	(1) sample2 2010-2015	(2) sample2 2015-2021	(3) sample2 2010-2015	(4) sample2 2015-2021	(5) sample3 2010-2015	(6) sample3 2015-2021	(7) sample3 2010-2015	(8) sample3 2015-2021	(9) sample1 2010-2015	(10) sample1 2015-2021	(11) sample1 2010-2015	(12) sample1 2015-2021	(13) sample1 2010-2015	(14) sample1 2015-2021
Observations	4097	770	7606	2349	3945	738	7255	2132	382	79	382	79	406	103
BIC	40639.2	7917.7	79803.8	25296.9	38748.5	7458.9	75628.7	22658.5	2778.0	-1523.4	2610.6	.	3668.5	636.8
AIC	40430.7	7815.5	79491.6	25066.4	38553.8	7371.4	75332.5	22437.6	2710.9	-1530.5	2543.5	.	3572.3	628.9

p-values in parentheses
* p<0.10, ** p<0.05, *** p<0.01

Table S7: Detailed results by period - Sensitivity: borrower ID fixed effect

	(1) sample2 2010-2015	(2) sample2 2015-2021	(3) sample2 2010-2015	(4) sample2 2015-2021	(5) sample3 2010-2015	(6) sample3 2015-2021	(7) sample3 2010-2015	(8) sample3 2015-2021	(9) sample1 2010-2015	(10) sample1 2015-2021	(11) sample1 2010-2015	(12) sample1 2015-2021	(13) sample1 2010-2015	(14) sample1 2015-2021
CDP score	-3.708	223.8			-1.796	188.8								
	(0.354)	(0.192)			(0.679)	(0.143)								
Refinitiv environmental score			0.0221	-0.695			0.697	-0.302						
			(0.979)	(0.299)			(0.416)	(0.716)						
Relative EIV									-723.8	-3842.3***				
									(0.136)	(0.000)				
AER											-599.3	-123.0***		
											(0.136)	(0.000)		
Energy saving technologies													-13.97	-154.4***
													(0.282)	(0.001)
Loan amount	-6.566	-725.7	8.087	4.207	-5.010	-613.0	4.857	-1.756						
	(0.290)	(0.202)	(0.201)	(0.803)	(0.520)	(0.131)	(0.512)	(0.936)						
Tranche amount	-8.863	-30.29	-0.297	-1.056	-10.55	-35.54*	-0.664	0.776	1.502	0.0000279	1.502	7.10e-08	1.502	1.476
	(0.200)	(0.117)	(0.954)	(0.878)	(0.114)	(0.099)	(0.906)	(0.906)	(0.307)	(0.309)	(0.307)	(0.316)	(0.306)	(0.484)
Number of lenders	5.777	91.11	-12.16	22.10	9.099	93.67	-10.59	41.28	2.499	-520.1***	2.499	-520.1***	2.499	-437.4***
	(0.459)	(0.152)	(0.157)	(0.459)	(0.212)	(0.137)	(0.292)	(0.208)	(0.584)	(0.000)	(0.584)	(0.000)	(0.584)	(0.000)
Maturity	21.84**	44.99	17.39**	-6.851	22.67**	87.10	19.20**	-0.832	-12.39	-0.000328	-12.39	-0.0000124	-12.39	-37.50***
	(0.037)	(0.212)	(0.030)	(0.620)	(0.031)	(0.312)	(0.017)	(0.952)	(0.227)	(0.432)	(0.227)	(0.432)	(0.226)	(0.000)
Firm size	68.30*	2344.2*	-7.942	-19.02	103.6**	2105.5**	-20.00	-78.91**	36.38	-58017.6***	-398.7*	2671.8***	-153.3***	-1382.7***
	(0.081)	(0.079)	(0.826)	(0.586)	(0.016)	(0.034)	(0.565)	(0.046)	(0.505)	(0.000)	(0.094)	(0.000)	(0.000)	(0.000)
Leverage	11.22	3097.6	17.07	-63.33	25.14	2676.6*	22.99	-5.382	-326.8	149918.0***	-2023.5	-7172.1***	-1163.9***	3297.6***
	(0.686)	(0.115)	(0.387)	(0.421)	(0.399)	(0.065)	(0.253)	(0.938)	(0.210)	(0.000)	(0.146)	(0.000)	(0.000)	(0.000)
Profitability	222.2	2006.8*	80.98	-62.62	71.82	1717.6**	55.16	142.3	955.6	4249010.6***	-1853.5*	-202676.2***	138.0	70730.9***

	(1)	(2)	(3)	(4)	(5)	(6)	(7)	(8)	(9)	(10)	(11)	(12)	(13)	(14)
	sample2 2010-2015	sample2 2015-2021	sample2 2010-2015	sample2 2015-2021	sample3 2010-2015	sample3 2015-2021	sample3 2010-2015	sample3 2015-2021	sample1 2010-2015	sample1 2015-2021	sample1 2010-2015	sample1 2015-2021	sample1 2010-2015	sample1 2015-2021
	(0.211)	(0.072)	(0.258)	(0.797)	(0.561)	(0.045)	(0.597)	(0.593)	(0.307)	(0.000)	(0.052)	(0.000)	(0.352)	(0.000)
Collateral=1	13.33**	45.60	16.46*	58.69*	3.880	51.08	7.840	56.53**	-68.03	-180757.9***	10.90	8949.9***	95.88***	-3866.7***
	(0.025)	(0.464)	(0.095)	(0.064)	(0.553)	(0.410)	(0.383)	(0.012)	(0.344)	(0.000)	(0.578)	(0.000)	(0.000)	(0.000)
Second-hand price index									41.72	-249.5***	-347.6*	-102.7***	-33.21***	-90.96***
									(0.440)	(0.000)	(0.094)	(0.000)	(0.000)	(0.000)
Ships' size									48.45**	-329.8***	-585.1	-19.12***	23.07***	31.25***
									(0.010)	(0.000)	(0.151)	(0.000)	(0.001)	(0.004)
Short maturity=1									25.71*	-0.0000254	25.71*	-0.00000172	25.71*	-5.380***
									(0.053)	(0.432)	(0.053)	(0.432)	(0.052)	(0.000)
Project finance=1									23.22***		23.22***		23.22***	
									(0.000)		(0.000)		(0.000)	
SPV=1									399.8		-1082.2*		125.6	
									(0.282)		(0.083)		(0.228)	
Year FE	Yes	Yes	Yes	Yes	Yes	Yes	Yes	Yes	Yes	Yes	Yes	Yes	Yes	Yes
Borrower Country FE	Yes	Yes	Yes	Yes	Yes	Yes	Yes	Yes						
Repayment type	Yes	Yes	Yes	Yes	Yes	Yes	Yes	Yes	Yes	Yes	Yes	Yes	Yes	Yes
Shipping segment	No	No	No	No	No	No	No	No	Yes	Yes	Yes	Yes	Yes	Yes
Industry FE	No	No	No	No	No	No	No	No	No	No	No	No	No	No
Borrower FE	Yes	Yes	Yes	Yes	Yes	Yes	Yes	Yes	Yes	Yes	Yes	Yes	Yes	Yes
R-squared	0.951	0.975	0.886	0.930	0.958	0.976	0.896	0.943	0.997	1.000	0.997	1	0.997	0.994
Observations	4097	770	7606	2349	3945	738	7255	2132	384	79	384	79	408	103
BIC	38552.8	7502.8	75357.1	23604.4	36563.9	7178.5	71315.1	21068.8	2185.5	-1512.1	2185.5	.	2314.5	636.8
AIC	38407.5	7423.8	75169.8	23471.9	36425.7	7109.5	71135.9	20944.2	2169.7	-1524.0	2169.7	.	2298.4	628.9

p-values in parentheses
* p<0.10, ** p<0.05, *** p<0.01

Table S8: Detailed results with Paris Agreement dummy. Central model

	(1)	(2)	(3)	(4)	(5)	(6)	(7)	(8)	(9)	(10)	(11)	(12)	(13)	(14)
	sample2 2010-2021	sample2 2010-2021	sample2 2010-2021	sample2 2010-2021	sample3 2010-2021	sample3 2010-2021	sample3 2010-2021	sample3 2010-2021	sample1 2010-2021	sample1 2010-2021	sample1 2010-2021	sample1 2010-2021	sample1 2010-2021	sample1 2010-2021
CDP score	-3.179	0.567			-2.916	1.292								
	(0.587)	(0.912)			(0.615)	(0.801)								
Post 2015 dummy=1 # CDP score		-35.37**				-48.65***								
		(0.027)				(0.010)								

	(1) sample2 2010-2021	(2) sample2 2010-2021	(3) sample2 2010-2021	(4) sample2 2010-2021	(5) sample3 2010-2021	(6) sample3 2010-2021	(7) sample3 2010-2021	(8) sample3 2010-2021	(9) sample1 2010-2021	(10) sample1 2010-2021	(11) sample1 2010-2021	(12) sample1 2010-2021	(13) sample1 2010-2021	(14) sample1 2010-2021
Refinitiv environmental score			-0.238	-0.213			-0.0920	-0.0174						
			(0.471)	(0.550)			(0.809)	(0.967)						
Post 2015 dummy=1 # Refinitiv environmental score				-0.165				-0.555						
				(0.717)				(0.302)						
Relative EIV									-3.159	-55.42				
									(0.930)	(0.231)				
Post 2015 dummy=1 # Relative EIV										142.5				
										(0.116)				
AER											-21.59	-58.49		
											(0.274)	(0.202)		
Post 2015 dummy=1 # AER												47.71		
												(0.343)		
Energy saving technologies													-5.759	14.89
													(0.825)	(0.698)
Post 2015 dummy=1 # Energy saving technologies														-50.87
														(0.338)
Loan amount	7.135	4.891	-7.408	-7.404	9.048	5.679	-5.323	-4.977						
	(0.493)	(0.571)	(0.193)	(0.193)	(0.403)	(0.489)	(0.386)	(0.417)						
Tranche amount	-5.180	-5.382	-2.757	-2.715	-3.525	-3.444	-2.102	-1.925	7.467	6.808	7.404	7.096	12.28	12.63
	(0.390)	(0.360)	(0.318)	(0.334)	(0.577)	(0.576)	(0.500)	(0.555)	(0.239)	(0.202)	(0.228)	(0.214)	(0.175)	(0.165)
Number of lenders	2.510	1.698	1.284	1.376	0.930	2.026	0.491	0.798	0.0136	-2.204	2.308	0.477	-2.193	-1.484
	(0.847)	(0.890)	(0.886)	(0.878)	(0.945)	(0.875)	(0.959)	(0.933)	(0.999)	(0.821)	(0.803)	(0.963)	(0.843)	(0.893)
Maturity	28.36***	26.80***	15.65**	15.50**	26.30***	24.13**	14.31**	13.64**	-29.27	-33.99	-33.98	-38.08*	21.72	19.72
	(0.005)	(0.007)	(0.022)	(0.024)	(0.007)	(0.011)	(0.031)	(0.043)	(0.152)	(0.114)	(0.122)	(0.081)	(0.376)	(0.403)
Firm size	-21.21	-18.69	-9.429	-9.179	-24.21	-22.59	-13.15*	-12.95*	-43.54***	-36.87***	-42.28***	-38.24***	-48.27***	-50.89***
	(0.151)	(0.184)	(0.171)	(0.184)	(0.119)	(0.115)	(0.095)	(0.099)	(0.001)	(0.005)	(0.002)	(0.006)	(0.000)	(0.000)
Leverage	22.00	25.95	39.59***	39.28***	25.58	28.79	37.55***	36.50***	37.34	88.61*	33.73	52.41	8.351	-2.541
	(0.360)	(0.261)	(0.002)	(0.002)	(0.301)	(0.212)	(0.003)	(0.005)	(0.467)	(0.073)	(0.499)	(0.258)	(0.877)	(0.966)
Profitability	80.44	67.61	-259.9*	-256.5*	139.4	127.3	-346.9**	-341.3**	-120.9	-146.9	-115.9	-108.7	-146.4	-139.7
	(0.706)	(0.743)	(0.056)	(0.064)	(0.494)	(0.510)	(0.025)	(0.029)	(0.224)	(0.132)	(0.226)	(0.280)	(0.164)	(0.198)

	(1) sample2 2010-2021	(2) sample2 2010-2021	(3) sample2 2010-2021	(4) sample2 2010-2021	(5) sample3 2010-2021	(6) sample3 2010-2021	(7) sample3 2010-2021	(8) sample3 2010-2021	(9) sample1 2010-2021	(10) sample1 2010-2021	(11) sample1 2010-2021	(12) sample1 2010-2021	(13) sample1 2010-2021	(14) sample1 2010-2021
Collateral=1	116.6**	112.6**	79.05***	79.83***	115.6**	110.8**	78.89***	80.42***	33.71**	37.10***	35.48*	38.79*	2.212	-8.526
	(0.018)	(0.017)	(0.004)	(0.003)	(0.019)	(0.017)	(0.005)	(0.005)	(0.046)	(0.002)	(0.066)	(0.056)	(0.917)	(0.771)
Second-hand price index									40.21***	40.74***	39.67***	42.22***	16.30	14.56
									(0.001)	(0.001)	(0.001)	(0.001)	(0.170)	(0.221)
Ships' size									-17.92	-33.07	-19.34	-21.40*	12.81	16.13
									(0.162)	(0.125)	(0.117)	(0.098)	(0.508)	(0.403)
Short maturity=1									47.08	36.28	49.39	40.91	67.66	67.88
									(0.224)	(0.335)	(0.215)	(0.308)	(0.196)	(0.195)
Project finance=1									13.54	13.96	13.12	11.03	-1.973	-0.100
									(0.274)	(0.223)	(0.301)	(0.413)	(0.918)	(0.995)
SPV=1									85.15**	85.92**	58.35*	73.71**	27.01	27.76
									(0.015)	(0.023)	(0.060)	(0.034)	(0.689)	(0.676)
Year FE	Yes	Yes	Yes	Yes	Yes	Yes	Yes	Yes	Yes	Yes	Yes	Yes	Yes	Yes
Borrower Country FE	Yes	Yes	Yes	Yes	Yes	Yes	Yes	Yes	Yes	Yes	Yes	Yes	Yes	Yes
Repayment type	Yes	Yes	Yes	Yes	Yes	Yes	Yes	Yes	Yes	Yes	Yes	Yes	Yes	Yes
Shipping segment	No	No	No	No	No	No	No	No	Yes	Yes	Yes	Yes	Yes	Yes
Industry FE	No	No	No	No	No	No	No	No	No	No	No	No	No	No
Borrower FE	No	No	No	No	No	No	No	No	No	No	No	No	No	No
R-squared	0.804	0.812	0.688	0.688	0.819	0.830	0.700	0.701	0.912	0.919	0.915	0.916	0.869	0.871
Observations	4867	4867	9955	9955	4683	4683	9387	9387	463	463	463	463	511	511
BIC	53654.0	53461.4	110485.7	110489.7	51355.3	51050.4	104012.6	103977.2	4309.0	4274.7	4297.0	4286.9	5017.9	5016.9
AIC	53400.9	53201.8	110139.9	110136.6	51110.1	50798.8	103683.8	103641.3	4184.9	4150.6	4172.9	4162.8	4873.9	4868.7

p-values in parentheses
* p<0.10, ** p<0.05, *** p<0.01

Table S9: Detailed results with Paris Agreement dummy. Sensitivity: industry fixed effect

	(1) sample2 2010-2021	(2) sample2 2010-2021	(3) sample2 2010-2021	(4) sample2 2010-2021	(5) sample3 2010-2021	(6) sample3 2010-2021	(7) sample3 2010-2021	(8) sample3 2010-2021	(9) sample1 2010-2021	(10) sample1 2010-2021	(11) sample1 2010-2021	(12) sample1 2010-2021	(13) sample1 2010-2021	(14) sample1 2010-2021
CDP score	-5.294	-3.177			-6.692	-4.935								
	(0.387)	(0.552)			(0.285)	(0.370)								
Post 2015 dummy=1 # CDP score		-23.93				-32.11*								
		(0.152)				(0.094)								
Refinitiv environmental score			-0.693**	-0.585			-0.669*	-0.582						
			(0.041)	(0.118)			(0.090)	(0.171)						

	(1)	(2)	(3)	(4)	(5)	(6)	(7)	(8)	(9)	(10)	(11)	(12)	(13)	(14)
	sample2 2010-2021	sample2 2010-2021	sample2 2010-2021	sample2 2010-2021	sample3 2010-2021	sample3 2010-2021	sample3 2010-2021	sample3 2010-2021	sample1 2010-2021	sample1 2010-2021	sample1 2010-2021	sample1 2010-2021	sample1 2010-2021	sample1 2010-2021
Post 2015 dummy=1 # Refinitiv environmental score				-0.773*				-1.248**						
				(0.084)				(0.013)						
Relative EIV									-35.23	-8.467				
									(0.135)	(0.792)				
Post 2015 dummy=1 # Relative EIV										-134.6				
										(0.314)				
AER											-34.18**	1.924		
											(0.043)	(0.957)		
Post 2015 dummy=1 # AER												-46.03		
												(0.271)		
Energy saving technologies													2.507	21.22
													(0.941)	(0.587)
Post 2015 dummy=1 # Energy saving technologies														-95.63
														(0.115)
Loan amount	19.71*	17.09**	6.382	6.356	21.06**	17.70**	6.373	6.907						
	(0.059)	(0.047)	(0.275)	(0.274)	(0.045)	(0.026)	(0.324)	(0.277)						
Tranche amount	3.263	3.437	-1.683	-1.412	2.727	2.914	-1.692	-1.249	-0.0529	-1.272	-1.084	-1.433	7.385	6.304
	(0.708)	(0.690)	(0.588)	(0.661)	(0.764)	(0.747)	(0.629)	(0.739)	(0.961)	(0.443)	(0.373)	(0.233)	(0.201)	(0.232)
Number of lenders	-15.39	-13.46	-10.29	-9.665	-17.51	-14.48	-10.52	-10.40	3.157	11.21	8.145	10.45	-7.648	-4.638
	(0.148)	(0.227)	(0.216)	(0.243)	(0.123)	(0.230)	(0.237)	(0.239)	(0.562)	(0.283)	(0.162)	(0.132)	(0.368)	(0.586)
Maturity	25.15**	24.42**	15.87***	15.29**	21.15**	20.15*	14.87**	13.99**	-35.41*	-37.09*	-38.71*	-34.58	7.189	4.207
	(0.020)	(0.026)	(0.009)	(0.015)	(0.044)	(0.057)	(0.011)	(0.021)	(0.070)	(0.055)	(0.074)	(0.126)	(0.744)	(0.848)
Firm size	-20.49	-19.95	-16.08**	-16.09**	-17.65	-15.15	-16.37**	-16.18**	5.154	15.41	7.354	5.219	-18.82	-14.68
	(0.131)	(0.123)	(0.026)	(0.025)	(0.206)	(0.250)	(0.035)	(0.032)	(0.610)	(0.144)	(0.405)	(0.599)	(0.231)	(0.308)
Leverage	2.575	3.833	29.89**	28.37**	11.54	16.04	29.55**	27.25*	102.9***	97.59***	115.4***	96.68**	-3.537	17.00
	(0.911)	(0.866)	(0.037)	(0.047)	(0.633)	(0.509)	(0.049)	(0.070)	(0.006)	(0.009)	(0.003)	(0.017)	(0.954)	(0.784)
Profitability	-281.1*	-279.8**	-228.1**	-215.9*	-185.7	-161.8	-273.4**	-254.8**	138.6	191.3**	116.2	130.5*	89.97	82.75
	(0.052)	(0.042)	(0.039)	(0.055)	(0.268)	(0.287)	(0.029)	(0.043)	(0.111)	(0.026)	(0.147)	(0.055)	(0.534)	(0.568)
Collateral=1	123.2***	120.0***	85.41***	89.05***	127.0***	123.5***	87.89***	92.14***	55.48***	58.26***	59.53***	53.00***	48.11	34.29
	(0.006)	(0.006)	(0.001)	(0.001)	(0.006)	(0.006)	(0.002)	(0.001)	(0.000)	(0.000)	(0.000)	(0.000)	(0.110)	(0.354)

	(1) sample2 2010-2021	(2) sample2 2010-2021	(3) sample2 2010-2021	(4) sample2 2010-2021	(5) sample3 2010-2021	(6) sample3 2010-2021	(7) sample3 2010-2021	(8) sample3 2010-2021	(9) sample1 2010-2021	(10) sample1 2010-2021	(11) sample1 2010-2021	(12) sample1 2010-2021	(13) sample1 2010-2021	(14) sample1 2010-2021
Second-hand price index									45.82***	41.65***	43.17***	40.57***	31.80***	26.78**
									(0.000)	(0.000)	(0.000)	(0.000)	(0.006)	(0.042)
Ships' size									9.288	6.100	-6.035	-5.524	55.36***	51.96***
									(0.622)	(0.746)	(0.770)	(0.800)	(0.001)	(0.004)
Short maturity=1									29.10	34.94	35.04	41.85	64.29	63.05
									(0.380)	(0.335)	(0.328)	(0.304)	(0.172)	(0.187)
Project finance=1									19.45***	23.48***	25.29***	29.80***	4.940	11.54
									(0.010)	(0.000)	(0.000)	(0.000)	(0.732)	(0.311)
SPV=1									35.18	-5.932	-14.89	-13.44	28.79	15.46
									(0.230)	(0.891)	(0.677)	(0.707)	(0.689)	(0.818)
Year FE	Yes	Yes	Yes	Yes	Yes	Yes	Yes	Yes	Yes	Yes	Yes	Yes	Yes	Yes
Borrower Country FE	Yes	Yes	Yes	Yes	Yes	Yes	Yes	Yes	Yes	Yes	Yes	Yes	Yes	Yes
Repayment type	Yes	Yes	Yes	Yes	Yes	Yes	Yes	Yes	Yes	Yes	Yes	Yes	Yes	Yes
Shipping segment	No	No	No	No	No	No	No	No	Yes	Yes	Yes	Yes	Yes	Yes
Industry FE	Yes	Yes	Yes	Yes	Yes	Yes	Yes	Yes	Yes	Yes	Yes	Yes	Yes	Yes
Borrower FE	No	No	No	No	No	No	No	No	No	No	No	No	No	No
R-squared	0.863	0.866	0.733	0.736	0.872	0.876	0.744	0.750	0.956	0.958	0.959	0.960	0.912	0.915
Observations	4867	4867	9955	9955	4683	4683	9387	9387	461	461	461	461	509	509
BIC	51923.9	51827.0	109001.6	108900.1	49738.1	49583.0	102588.6	102370.9	3932.8	3911.7	3902.2	3888.0	4771.6	4754.3
AIC	51651.3	51548.0	108598.1	108489.4	49480.0	49318.5	102202.6	101977.8	3833.6	3812.5	3803.0	3788.8	4640.4	4623.1

p-values in parentheses
* p<0.10, ** p<0.05, *** p<0.01

Table S10: Detailed results with Paris Agreement dummy. Sensitivity: borrower ID fixed effect

	(1) sample2 2010-2021	(2) sample2 2010-2021	(3) sample2 2010-2021	(4) sample2 2010-2021	(5) sample3 2010-2021	(6) sample3 2010-2021	(7) sample3 2010-2021	(8) sample3 2010-2021	(9) sample1 2010-2021	(10) sample1 2010-2021	(11) sample1 2010-2021	(12) sample1 2010-2021	(13) sample1 2010-2021	(14) sample1 2010-2021
CDP score	-0.135	3.648			4.805	7.096								
	(0.983)	(0.517)			(0.420)	(0.203)								
Post 2015 dummy=1 # CDP score		-34.67**				-31.98**								
		(0.026)				(0.038)								
Refinitiv environmental score			-0.00132	0.368			0.144	0.552						
			(0.997)	(0.444)			(0.752)	(0.316)						
Post 2015 dummy=1 # Refinitiv environmental score				-1.662***				-1.675***						
				(0.000)				(0.000)						

	(1)	(2)	(3)	(4)	(5)	(6)	(7)	(8)	(9)	(10)	(11)	(12)	(13)	(14)
	sample2	sample2	sample2	sample2	sample3	sample3	sample3	sample3	sample1	sample1	sample1	sample1	sample1	sample1
	2010-2021	2010-2021	2010-2021	2010-2021	2010-2021	2010-2021	2010-2021	2010-2021	2010-2021	2010-2021	2010-2021	2010-2021	2010-2021	2010-2021
Relative EIV									-530.8	-680.3***				
									(0.243)	(0.008)				
Post 2015 dummy=1 # Relative EIV										1441.5***				
										(0.000)				
AER											34.28	-21.25		
											(0.622)	(0.799)		
Post 2015 dummy=1 # AER												198.2*		
												(0.081)		
Energy saving technologies													-90.81**	-158.2***
													(0.040)	(0.000)
Post 2015 dummy=1 # Energy saving technologies														262.6
														(0.207)
Loan amount	15.20**	14.67*	7.033	7.094	12.92	14.77	4.465	5.001						
	(0.050)	(0.064)	(0.180)	(0.210)	(0.141)	(0.121)	(0.458)	(0.443)						
Tranche amount	-6.478	-5.980	-0.497	-0.626	-9.438*	-9.072*	-1.097	-0.905	-0.430	-0.431	-0.300	-0.203	-0.477	-0.381
	(0.249)	(0.297)	(0.897)	(0.880)	(0.080)	(0.098)	(0.791)	(0.841)	(0.744)	(0.730)	(0.821)	(0.865)	(0.738)	(0.770)
Number of lenders	4.941	9.094	-5.489	-5.506	11.72	14.27	-0.933	-1.913	-1.708	-1.602	-1.800	-1.607	-1.701	-1.521
	(0.599)	(0.360)	(0.508)	(0.516)	(0.255)	(0.180)	(0.921)	(0.845)	(0.827)	(0.838)	(0.818)	(0.837)	(0.824)	(0.841)
Maturity	24.69***	23.54***	16.78***	15.18**	25.29***	23.92***	18.92***	17.24***	-45.93	-46.23	-46.10	-45.53	-45.26*	-44.17*
	(0.005)	(0.007)	(0.008)	(0.021)	(0.005)	(0.007)	(0.002)	(0.008)	(0.130)	(0.127)	(0.127)	(0.131)	(0.068)	(0.070)
Firm size	-74.12	-74.39	-86.17**	-95.88***	-53.43	-59.48	-96.90***	-106.4***	-301.9	-70.47	-200.3	-140.8	266.6	671.3**
	(0.269)	(0.214)	(0.011)	(0.003)	(0.435)	(0.349)	(0.004)	(0.001)	(0.173)	(0.706)	(0.493)	(0.633)	(0.562)	(0.033)
Leverage	116.8**	96.16**	54.97***	47.87**	103.2*	84.26	62.70***	56.81***	-619.0	1788.2**	476.8	742.8	-1567.5***	-1008.5*
	(0.031)	(0.034)	(0.006)	(0.013)	(0.093)	(0.110)	(0.001)	(0.002)	(0.523)	(0.017)	(0.395)	(0.346)	(0.006)	(0.061)
Profitability	139.8	127.1	-73.63	-41.67	-46.15	-18.07	-123.3	-66.80	950.5	320.2	55.59	-188.0	6.036	-856.4*
	(0.174)	(0.146)	(0.404)	(0.601)	(0.737)	(0.886)	(0.218)	(0.452)	(0.228)	(0.444)	(0.456)	(0.314)	(0.993)	(0.077)
Collateral=1	66.05*	62.23*	53.20**	61.32***	56.46*	56.55*	46.90**	55.59**	57.05	-64.23	116.2*	88.80	-30.49	-202.8*
	(0.054)	(0.053)	(0.014)	(0.006)	(0.067)	(0.067)	(0.029)	(0.014)	(0.481)	(0.421)	(0.091)	(0.168)	(0.850)	(0.054)
Second-hand price index									8.430	56.94***	24.68	18.31	28.89*	29.12*
									(0.593)	(0.000)	(0.143)	(0.387)	(0.084)	(0.085)

	(1) sample2 2010-2021	(2) sample2 2010-2021	(3) sample2 2010-2021	(4) sample2 2010-2021	(5) sample3 2010-2021	(6) sample3 2010-2021	(7) sample3 2010-2021	(8) sample3 2010-2021	(9) sample1 2010-2021	(10) sample1 2010-2021	(11) sample1 2010-2021	(12) sample1 2010-2021	(13) sample1 2010-2021	(14) sample1 2010-2021
Ships' size									-18.27	77.63***	40.44	54.01	34.93***	-4.349
									(0.707)	(0.001)	(0.305)	(0.218)	(0.000)	(0.877)
Short maturity=1									47.18	46.82	48.28	49.80	46.55	48.69
									(0.260)	(0.258)	(0.257)	(0.246)	(0.272)	(0.261)
Project finance=1									25.38***	25.39***	25.18***	25.07***	25.45***	25.33***
									(0.000)	(0.000)	(0.000)	(0.000)	(0.000)	(0.000)
SPV=1									254.7**	524.6***	176.1	436.1	439.4*	118.5
									(0.012)	(0.000)	(0.202)	(0.153)	(0.067)	(0.637)
Year FE	Yes	Yes	Yes	Yes	Yes	Yes	Yes	Yes	Yes	Yes	Yes	Yes	Yes	Yes
Borrower Country FE	Yes	Yes	Yes	Yes	Yes	Yes	Yes	Yes	Yes	Yes	Yes	Yes	Yes	Yes
Repayment type	Yes	Yes	Yes	Yes	Yes	Yes	Yes	Yes	Yes	Yes	Yes	Yes	Yes	Yes
Shipping segment	No	No	No	No	No	No	No	No	Yes	Yes	Yes	Yes	Yes	Yes
Industry FE	No	No	No	No	No	No	No	No	No	No	No	No	No	No
Borrower FE	Yes	Yes	Yes	Yes	Yes	Yes	Yes	Yes	Yes	Yes	Yes	Yes	Yes	Yes
R-squared	0.926	0.929	0.843	0.850	0.931	0.934	0.854	0.861	0.980	0.980	0.980	0.980	0.982	0.983
Observations	4867	4867	9955	9955	4683	4683	9387	9387	463	463	463	463	511	511
BIC	48861.0	48648.9	103516.3	103062.7	46733.3	46561.4	97133.8	96680.3	3494.7	3485.0	3500.3	3489.8	3828.9	3811.0
AIC	48672.8	48454.2	103264.1	102803.3	46552.7	46374.3	96890.8	96430.2	3461.6	3451.9	3467.2	3456.7	3795.0	3785.6

p-values in parentheses
* p<0.10, ** p<0.05, *** p<0.01

Table S11: Detailed results, effect of the Poseidon Principles. Central model.

	(1) sample2 2010-2021	(2) sample2 2010-2021	(3) sample3 2010-2021	(4) sample3 2010-2021	(5) sample1 2010-2021	(6) sample1 2010-2021	(7) sample1 2010-2021
CDP score	-2.367		-2.153				
	(0.676)		(0.702)				
Poseidon Principles signatory=1 # CDP score	-56.02***		-54.02***				
	(0.001)		(0.001)				
Refinitiv environmental score		-0.249		-0.107			
		(0.445)		(0.777)			
Poseidon Principles signatory=1 # Refinitiv environmental score		1.285		1.077			
		(0.108)		(0.379)			
Relative EIV					-13.59		
					(0.718)		
Poseidon Principles signatory=1 # Relative EIV					55.29		
					(0.126)		
AER						-23.55	

	(1) sample2 2010-2021	(2) sample2 2010-2021	(3) sample3 2010-2021	(4) sample3 2010-2021	(5) sample1 2010-2021	(6) sample1 2010-2021	(7) sample1 2010-2021
						(0.234)	
Poseidon Principles signatory=1 # AER						-4.512	
						(0.529)	
Energy saving technologies							-7.304
							(0.807)
Poseidon Principles signatory=1 # Energy saving technologies							4.814
							(0.910)
Loan amount	5.255	-7.449	7.117	-5.540			
	(0.588)	(0.189)	(0.482)	(0.366)			
Tranche amount	-5.597	-2.948	-3.932	-2.221	7.277	7.146	12.17
	(0.341)	(0.290)	(0.526)	(0.478)	(0.235)	(0.236)	(0.185)
Number of lenders	3.446	1.481	1.857	0.536	0.116	3.040	-2.217
	(0.787)	(0.868)	(0.889)	(0.955)	(0.990)	(0.742)	(0.842)
Maturity	28.60***	15.78**	26.55***	14.49**	-30.14	-34.32	21.77
	(0.004)	(0.019)	(0.006)	(0.029)	(0.140)	(0.121)	(0.376)
Firm size	-19.36	-9.546	-22.12	-12.74*	-42.03***	-41.08***	-47.90***
	(0.171)	(0.159)	(0.139)	(0.099)	(0.001)	(0.002)	(0.000)
Leverage	22.94	39.18***	26.69	37.57***	47.63	40.60	10.61
	(0.338)	(0.002)	(0.280)	(0.003)	(0.331)	(0.418)	(0.866)
Profitability	85.58	-258.4*	144.0	-345.4**	-127.7	-122.7	-147.9
	(0.683)	(0.059)	(0.472)	(0.026)	(0.193)	(0.195)	(0.170)
Collateral=1	114.5**	78.29***	113.3**	78.18***	33.87**	36.40*	2.437
	(0.020)	(0.004)	(0.021)	(0.007)	(0.031)	(0.052)	(0.911)
Second-hand price index					39.53***	38.72***	16.29
					(0.001)	(0.001)	(0.169)
Ships' size					-21.61	-23.05*	12.42
					(0.122)	(0.091)	(0.524)
Short maturity=1					45.47	49.75	67.51
					(0.237)	(0.216)	(0.200)
Project finance=1					14.00	14.23	-1.802
					(0.243)	(0.245)	(0.925)
SPV=1					80.20**	47.67	26.94
					(0.025)	(0.140)	(0.691)
Year FE	Yes	Yes	Yes	Yes	Yes	Yes	Yes
Borrower Country FE	Yes	Yes	Yes	Yes	Yes	Yes	Yes

	(1)<br>sample2 2010-2021	(2)<br>sample2 2010-2021	(3)<br>sample3 2010-2021	(4)<br>sample3 2010-2021	(5)<br>sample1 2010-2021	(6)<br>sample1 2010-2021	(7)<br>sample1 2010-2021
Repayment type	Yes	Yes	Yes	Yes	Yes	Yes	Yes
Shipping segment	No	No	No	No	Yes	Yes	Yes
Industry FE	No	No	No	No	No	No	No
Borrower FE	No	No	No	No	No	No	No
R-squared	0.809	0.690	0.823	0.701	0.914	0.916	0.869
Observations	4867	9955	4683	9387	463	463	511
BIC	53561.7	110453.0	51265.3	103986.7	4301.5	4292.0	5023.9
AIC	53295.6	110092.7	51007.2	103643.6	4177.4	4167.8	4875.6

p-values in parentheses
* p<0.10, ** p<0.05, *** p<0.01

Table S13: Detailed results, effect of the Poseidon Principles. Sensitivity: industry fixed effects

	(1)<br>sample2 2010-2021	(2)<br>sample2 2010-2021	(3)<br>sample3 2010-2021	(4)<br>sample3 2010-2021	(5)<br>sample1 2010-2021	(6)<br>sample1 2010-2021	(7)<br>sample1 2010-2021
CDP score	-2.367		-2.153				
	(0.676)		(0.702)				
Poseidon Principles signatory=1 # CDP score	-56.02***		-54.02***				
	(0.001)		(0.001)				
Refinitiv environmental score		-0.249		-0.107			
		(0.445)		(0.777)			
Poseidon Principles signatory=1 # Refinitiv environmental score		1.285		1.077			
		(0.108)		(0.379)			
Relative EIV					-2.392		
					(0.939)		
Poseidon Principles signatory=1 # Relative EIV					50.15*		
					(0.096)		
AER						7.519	
						(0.649)	
Poseidon Principles signatory=1 # AER						1.345	
						(0.775)	
Energy saving technologies							-14.07
							(0.641)
Poseidon Principles signatory=1 # Energy saving technologies							3.682
							(0.931)
Loan amount	5.255	-7.449	7.117	-5.540			
	(0.588)	(0.189)	(0.482)	(0.366)			

	(1) sample2 2010-2021	(2) sample2 2010-2021	(3) sample3 2010-2021	(4) sample3 2010-2021	(5) sample1 2010-2021	(6) sample1 2010-2021	(7) sample1 2010-2021
Tranche amount	-5.597	-2.948	-3.932	-2.221	5.944	6.112	11.89
	(0.341)	(0.290)	(0.526)	(0.478)	(0.232)	(0.230)	(0.192)
Number of lenders	3.446	1.481	1.857	0.536	4.864	4.449	-4.414
	(0.787)	(0.868)	(0.889)	(0.955)	(0.513)	(0.551)	(0.710)
Maturity	28.60***	15.78**	26.55***	14.49**	-67.57**	-68.33**	18.49
	(0.004)	(0.019)	(0.006)	(0.029)	(0.019)	(0.014)	(0.439)
Firm size	-19.36	-9.546	-22.12	-12.74*	-43.28***	-44.46***	-48.97***
	(0.171)	(0.159)	(0.139)	(0.099)	(0.000)	(0.000)	(0.000)
Leverage	22.94	39.18***	26.69	37.57***	128.1***	127.2***	19.46
	(0.338)	(0.002)	(0.280)	(0.003)	(0.002)	(0.004)	(0.746)
Profitability	85.58	-258.4*	144.0	-345.4**	-22.53	-11.88	-133.4
	(0.683)	(0.059)	(0.472)	(0.026)	(0.800)	(0.886)	(0.239)
Collateral=1	114.5**	78.29***	113.3**	78.18***	41.68***	41.39**	1.911
	(0.020)	(0.004)	(0.021)	(0.007)	(0.007)	(0.016)	(0.929)
Second-hand price index					40.92***	41.67***	17.37
					(0.000)	(0.000)	(0.169)
Ships' size					-32.26***	-29.94**	17.34
					(0.006)	(0.012)	(0.427)
Ships' age					-9.496***	-10.05***	-1.258
					(0.000)	(0.000)	(0.108)
Short maturity=1					51.68	51.93	67.26
					(0.224)	(0.219)	(0.199)
Project finance=1					29.94***	30.18***	0.154
					(0.003)	(0.002)	(0.993)
SPV=1					38.77	48.38	34.84
					(0.264)	(0.191)	(0.629)
Year FE	Yes	Yes	Yes	Yes	Yes	Yes	Yes
Borrower Country FE	Yes	Yes	Yes	Yes	Yes	Yes	Yes
Repayment type	Yes	Yes	Yes	Yes	Yes	Yes	Yes
Shipping segment	No	No	No	No	Yes	Yes	Yes
Industry FE	Yes	Yes	Yes	Yes	Yes	Yes	Yes
Borrower FE	No	No	No	No	No	No	No
R-squared	0.809	0.690	0.823	0.701	0.933	0.933	0.871
Observations	4867	9955	4683	9387	463	463	511
BIC	53561.7	110453.0	51265.3	103986.7	4183.2	4187.6	5023.0
AIC	53295.6	110092.7	51007.2	103643.6	4059.0	4063.4	4870.5

p-values in parentheses

* p<0.10, ** p<0.05, *** p<0.01

Table S14: Detailed results, effect of the Poseidon Principles. Sensitivity: borrower ID fixed effect

	(1) sample2 2010-2021	(2) sample2 2010-2021	(3) sample3 2010-2021	(4) sample3 2010-2021	(5) sample1 2010-2021	(6) sample1 2010-2021	(7) sample1 2010-2021
CDP score	-0.0967		4.692				
	(0.987)		(0.430)				
Poseidon Principles signatory=1 # CDP score	-26.83***		-25.79***				
	(0.002)		(0.003)				
Refinitiv environmental score		-0.0228		0.149			
		(0.956)		(0.741)			
Poseidon Principles signatory=1 # Refinitiv environmental score		0.690		-0.970			
		(0.491)		(0.261)			
Relative EIV					-530.8		
					(0.244)		
Poseidon Principles signatory=1 # Relative EIV					-1.32e-10		
					(0.270)		
AER						34.28	
						(0.623)	
Poseidon Principles signatory=1 # AER						3.00e-12	
						(0.853)	
Energy saving technologies							-90.81**
							(0.040)
Poseidon Principles signatory=1 # Energy saving technologies							-8.44e-11
							(0.496)
Loan amount	14.65*	6.914	12.58	4.461			
	(0.060)	(0.187)	(0.154)	(0.458)			
Tranche amount	-6.589	-0.590	-9.515*	-1.040	-0.430	-0.300	-0.477
	(0.243)	(0.878)	(0.080)	(0.803)	(0.745)	(0.821)	(0.738)
Number of lenders	5.615	-5.176	12.21	-0.890	-1.708	-1.800	-1.701
	(0.546)	(0.531)	(0.231)	(0.924)	(0.827)	(0.819)	(0.825)
Maturity	24.82***	16.78***	25.35***	18.91***	-45.93	-46.10	-45.26*
	(0.005)	(0.008)	(0.005)	(0.002)	(0.131)	(0.128)	(0.069)
Firm size	-64.95	-84.04**	-45.06	-94.22***	-301.9	-200.3	266.6
	(0.306)	(0.012)	(0.491)	(0.004)	(0.174)	(0.494)	(0.563)
Leverage	109.7**	54.75***	96.66*	61.71***	-619.0	476.8	-1567.5***
	(0.033)	(0.005)	(0.098)	(0.001)	(0.524)	(0.396)	(0.006)

	(1)<br>sample2 2010-2021	(2)<br>sample2 2010-2021	(3)<br>sample3 2010-2021	(4)<br>sample3 2010-2021	(5)<br>sample1 2010-2021	(6)<br>sample1 2010-2021	(7)<br>sample1 2010-2021
Profitability	144.3	-69.70	-37.08	-122.8	950.5	55.59	6.036
	(0.153)	(0.418)	(0.786)	(0.218)	(0.229)	(0.458)	(0.993)
Collateral=1	64.98*	52.63**	55.64*	45.88**	57.05	116.2*	-30.49
	(0.054)	(0.013)	(0.067)	(0.030)	(0.482)	(0.092)	(0.851)
Second-hand price index					8.430	24.68	28.89*
					(0.594)	(0.144)	(0.084)
Ships' size					-18.27	40.44	34.93***
					(0.708)	(0.306)	(0.000)
Short maturity=1					47.18	48.28	46.55
					(0.261)	(0.259)	(0.273)
Project finance=1					25.38***	25.18***	25.45***
					(0.000)	(0.000)	(0.000)
SPV=1					254.7**	176.1	439.4*
					(0.012)	(0.203)	(0.068)
Year FE	Yes	Yes	Yes	Yes	Yes	Yes	Yes
Borrower Country FE	Yes	Yes	Yes	Yes	Yes	Yes	Yes
Repayment type	Yes	Yes	Yes	Yes	Yes	Yes	Yes
Shipping segment	No	No	No	No	Yes	Yes	Yes
Industry FE	No	No	No	No	No	No	No
Borrower FE	Yes	Yes	Yes	Yes	Yes	Yes	Yes
R-squared	0.926	0.844	0.932	0.855	0.980	0.980	0.982
Observations	4867	9955	4683	9387	463	463	511
BIC	48821.3	103503.0	46695.5	97097.5	3494.7	3500.3	3828.9
AIC	48620.2	103236.4	46501.9	96840.2	3461.6	3467.2	3795.0

p-values in parentheses

* p<0.10, ** p<0.05, *** p<0.01
